# Multi-Agent Deep Reinforcement Learning for Multi-Echelon Inventory Management

**DOI:** 10.1177/10591478241305863

**Published:** 2024-12-29

**Authors:** Xiaotian Liu, Ming Hu, Yijie Peng, Yaodong Yang

**Affiliations:** 1 Guanghua School of Management, 12465 Peking University, Beijing, China; 2 Rotman School of Management, University of Toronto, Toronto, ON, Canada; 3 PKU-Wuhan Institute for Artificial Intelligence, Guanghua School of Management, Xiangjiang Laboratory, 12465 Peking University, Beijing, China; 4 Institute for Artificial Intelligence, PKU-Wuhan Institute for Artificial Intelligence, 12465 Peking University, Beijing, China

**Keywords:** Multi-Echelon Inventory Management, Multi-Agent Reinforcement Learning, Bullwhip Effect

## Abstract

We apply heterogeneous-agent proximal policy optimization (HAPPO), a multi-agent deep reinforcement learning (MADRL) algorithm, to the decentralized multi-echelon inventory management problems in both a serial supply chain and a supply chain network. We also examine whether the upfront-only information-sharing mechanism used in MADRL helps alleviate the bullwhip effect. Our results show that policies constructed by HAPPO achieve lower overall costs than policies constructed by single-agent deep reinforcement learning and other heuristic policies. Also, the application of HAPPO results in a less significant bullwhip effect than policies constructed by single-agent deep reinforcement learning where information is not shared among actors. Somewhat surprisingly, compared to using the overall costs of the system as a minimization target for each actor, HAPPO achieves lower overall costs when the minimization target for each actor is a combination of its own costs and the overall costs of the system. Our results provide a new perspective on the benefit of information sharing inside the supply chain that helps alleviate the bullwhip effect and improve the overall performance of the system. Upfront information sharing and action coordination in model training among actors is essential, with the former even more essential, for improving a supply chain’s overall performance when applying MADRL. Neither actors being fully self-interested nor actors being fully system-focused leads to the best practical performance of policies learned and constructed by MADRL. Our results also verify MADRL’s potential in solving various multi-echelon inventory management problems with complex supply chain structures and in non-stationary market environments.

## 1. Introduction

The unpredictable outbreak of COVID-19 disrupts the global supply chain severely, causing tremendous economic loss. In the post-pandemic era, supply chain management (SCM) has been playing an increasingly critical role in global economics. Inventory management is central in SCM and has a significant impact on the service level and economic efficiency of a supply chain network (SCN) where multiple echelons are involved, and each echelon contains multiple parallel actors. Every actor in the SCN faces complicated inventory decision problems characteristic of lost sales, dual sourcing, and perishable inventory. For the coordination of multiple actors in a supply chain, the demand spikes caused by the pandemic amplify the demand distortion along the supply chain and aggravate the bullwhip effect in many businesses (Stank et al., 2021). The bullwhip effect describes a commonly observed phenomenon that orders faced by different entities from downstream to upstream of a supply chain have increasing variability (Lee et al., 1997), which would lead to significant overstock or backlogs and thereby harm the economic performance of the supply chain.

The literature on inventory management problems is rich, but most theoretical studies require strong assumptions such as independently and identically distributed (i.i.d.) demands and a simple SCN structure, which are typically unrealistic in the practice of SCM. To open up new opportunities for inventory management, artificial intelligence (AI), a disruptive technology for various areas, has attracted increasingly more research interests. As a pivotal technique in AI, reinforcement learning (RL) uses feedback obtained via interactions with the environment to learn the optimal policy for Markov decision processes (MDPs). Deep reinforcement learning (DRL) further improves the learning efficiency for RL by using neural networks (NNs) to parameterize the value function and the policy, which leads to many successful applications in various domains, including gaming, robotic manipulation, and self-driving cars.

The sequential decision problems in inventory management can usually be formulated as MDPs, where DRL naturally applies (Boute et al., 2022). Though current results have clearly demonstrated DRL’s potential in some complex inventory problems where calculating the exact optimal policy is intractable, information sharing and collaboration among multiple actors inside SCN remain unexplored. To apply DRL to inventory management problems with multiple actors, previous studies such as Gijsbrechts et al. (2021), Vanvuchelen et al. (2020), and Harsha et al. (2022) train a centralized agent to make decisions for all actors. The rationale behind this scheme relies heavily on a “central control tower” assumption, that is, there exists a central control tower that has access to all information in the supply chain and can always control every actor. Apparently, such an assumption rarely holds in the practice of SCM. Vosooghidizaji et al. (2020) summarized multiple strategic reasons that cause information asymmetry along the supply chain, such as the fear of losing competitive advantage, getting extra benefits, ensuring compatibility of information systems, and so on. In recent years, the digitization of a supply chain has made data leakage a critical issue that prevents participants of SCM from sharing real-time information. In 2004, Walmart announced not to share data even with some collaborating companies since it takes data as its private resources (Hays, 2004). Furthermore, the increasing number of cyberattacks has made cybersecurity a new concern that obstructs data and information sharing inside the supply chain. To ensure cybersecurity, both companies and governments, such as China, have put out specific rules that regulate the outflow of private data. All the aforementioned factors make it almost impossible to achieve complete information sharing among practical supply chains. How to construct DRL policies for multiple actors in a supply chain with practical information-sharing conditions remains unsolved in both the practical and academic worlds.

To fill such a research gap, we formulate a supply chain as a multi-agent system (MAS) and apply multi-agent DRL (MADRL) to the multi-echelon inventory management problems where multiple actors from different echelons are involved. As a subfield of DRL, MADRL focuses on coordinating a group of autonomous agents to achieve certain goals by interacting with a shared environment. A centralized training and decentralized execution (CTDE) scheme is characteristic of MADRL algorithms. Specifically, each actor learns a policy by resorting to all actors’ observations in the training stage while making decisions only based on their own observations in the execution stage. In the context of decentralized inventory management, CTDE creates a unique coordination mechanism between fully centralized and fully decentralized systems. Specifically, in an upfront training stage, each actor in the supply chain first learns to make coordinated ordering decisions under the knowledge of other actors’ information and then engages in the practice of decentralized inventory management where other actors’ information is not available. CTDE makes MADRL suited for multi-echelon inventory management problems because information sharing is required only in the training stage, so the scheme is implementable in practical supply chain settings. Participants of SCM can use and share historical data to conduct one-time centralized training. The trained models can then be deployed by different participants in a decentralized manner. Such a coordination mechanism may benefit the collaborating partners in a supply chain who have issues with sharing real-time data because of various reasons mentioned before. For example, it may help Amazon work better with its vendors and third-party sellers to improve the efficiency of the whole supply chain, given that each party manages only part of the chain. Meanwhile, it can be beneficial to some large retail or logistics companies that control multiple stages in a supply chain but find it challenging to synchronize data among multiple self-controlled stages in real time.

Our contributions can be summarized as follows:

(1) We provide the formulation of multi-echelon inventory management problems in a serial supply chain system and a supply chain network system as partially observable Markov games (POMGs) and apply MADRL to construct intelligent ordering policies.
(2) We investigate a unique form of training objective for each actor from both theoretical and numerical aspects. Compared to setting the target for each actor as minimizing the overall costs, the system achieves lower overall costs when each actor considers minimizing a combination of its own costs and the overall costs of the system.
(3) We experimentally verify that MADRL is superior in lowering the overall costs of the system, and the upfront-only information sharing in the training stage of MADRL is effective in alleviating the bullwhip effect.

## 2. Literature Review

### 2.1. Related Inventory Management Problems

The most classic inventory management literature considers a single-sourcing backlogged model with stationary customer demand and a deterministic lead time. With linear backlog and holding costs and in the absence of fixed order costs, the base stock policy has proved to be optimal for minimizing expected overall costs. If the fixed costs are also considered in the cost structure, the optimal policy becomes an ( s , S ) policy. With non-stationary customer demand, the optimal policies for the aforementioned two settings become non-stationary base stock and non-stationary ( s , S ), respectively. Deriving closed forms of the optimal parameters in non-stationary base stock and non-stationary ( s , S ) policies is typically intractable, which motivates researchers to develop heuristics to determine parameters in non-stationary policies. Specifically, Neale and Willems (2009) introduced a practical method based on the guaranteed service framework to determine the period-dependent base stock level for the non-stationary base stock policy. The base stock level changes by referring to the mean and variance of historical demands. Bollapragada and Morton (1999) introduced a well-acknowledged method to determine the period-dependent s and S parameters for non-stationary ( s , S ) policy in the presence of fixed costs. It first finds the optimal policy parameters, that is, s and S , under the case of stationary demands and then approximates the non-stationary problem at each period with a stationary problem.

**Figure 1.**
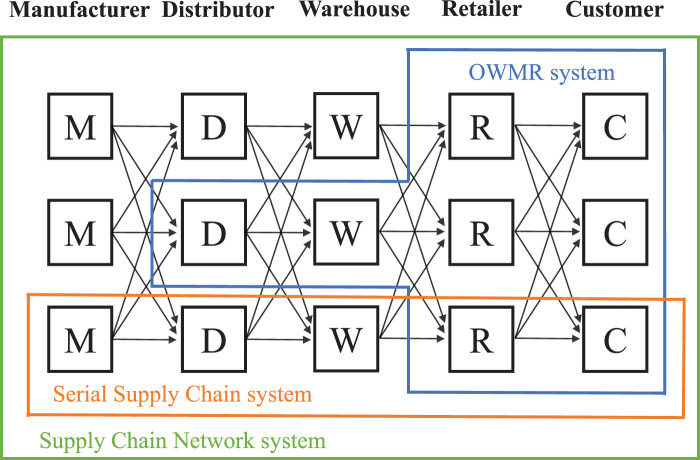
Three extensively studied systems in multi-echelon inventory management problem.

The dual-sourcing model complicates inventory management by assuming that an actor replenishes inventory from two sources: a normal source and an expedited source with a shorter lead time but a higher ordering cost. For the dual-souring inventory management under the assumption of backlog, Veeraraghavan and Scheller-Wolf (2008) proposed a dual index (DI) policy, where two inventory positions are maintained separately for normal and expedited orders. As a variant of DI, the capped dual index (CDI) policy proposed by Sun and Mieghem (2019) adds a cap on normal ordering. It is proven to be optimal with general lead times and stationary or non-stationary demand. Different from DI and CDI, the tailored base surge (TBS) policy proposed by Allon and Mieghem (2010) makes a constant order from the regular source and follows an ( s , S ) policy for expedited orders. Its asymptotic optimality in the scenario of a fixed lead time for the expedited source and a large lead time for the normal source is proved by Xin and Goldberg (2018). Gijsbrechts et al. (2021) formulate the dual-sourcing inventory management problem as an MDP and apply a popular DRL algorithm called asynchronous advantage actor critic (A3C) (Mnih et al., 2016). Their results show that the performance of A3C is comparable to TBS and DI but inferior to CDI with stationary customer demand.

Different from the above-mentioned problems that focus on a single actor in a supply chain, a multi-echelon inventory management problem involves multiple actors from different echelons. As shown in Figure 1, three systems have been studied extensively in the multi-echelon inventory management problem.

In the one-warehouse multi-retailer (OWMR) system, one warehouse is shared by several retailers with separate demands. Compared to the OWMR system, the serial supply chain system has a longer but thinner structure. The general supply chain network system further generalizes the structure of a supply chain by adding parallel actors into an echelon.

**Figure 2.**
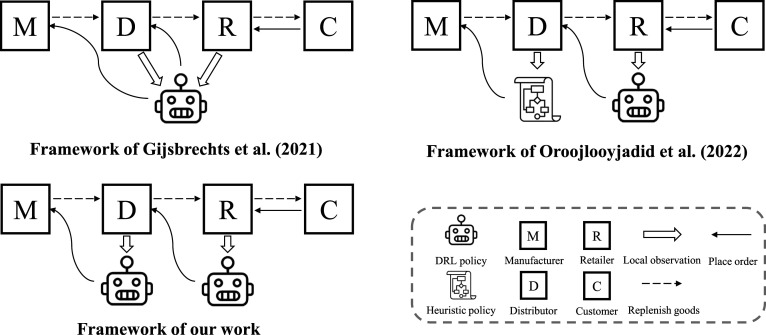
Comparison of our work and previous literature on applying DRL to multi-echelon inventory management problems.

Gijsbrechts et al. (2021) applied A3C to an OWMR system. Their result shows that A3C beats a base stock policy by 9%–12%. Harsha et al. (2022) introduced a deep-policy iteration RL method that outperforms several state-of-the-art RL algorithms and the ( s , S ) policy for a supply chain network system with a large combinatorial action space and state-dependent constraints. As mentioned, both of these two papers make a somewhat restrictive “central control tower” assumption and construct policies for all actors with a single DRL agent, which takes all actors’ observations as inputs and prescribes all actors’ actions. In contrast, Oroojlooyjadid et al. (2022) considered a decentralized serial supply chain system where actors make decisions only based on their local observations. Constructing DRL policies for multiple actors under this scenario is challenging because training multiple actors simultaneously makes the environment non-stationary. Due to this difficulty, the authors only construct the DRL policy for one actor in the supply chain, with the other actors following simple heuristic policies. To overcome the challenge, our work aims to construct DRL policies for all actors in a supply chain that are effective in lowering the overall costs and do not require full information sharing all the time as needed by Gijsbrechts et al. (2021). A comparison of Gijsbrechts et al. (2021), Oroojlooyjadid et al. (2022), and our work is visualized in Figure 2. Because of the unique upfront-only information-sharing mechanism, our MADRL-based method can be positioned between Gijsbrechts et al. (2021), which is a fully centralized method, and Oroojlooyjadid et al. (2022), which is a fully decentralized method. Moreover, we consider more general multi-echelon inventory management problems in both a serial supply chain system and a supply chain network system.

### 2.2. Bullwhip Effect

Introduced and studied first by Procter & Gamble (PG) in the 1990s, the bullwhip effect refers to the phenomenon that demand fluctuations increase from downstream to upstream in a supply chain. A further study by Lee et al. (1997) demonstrates how distorted information transmitted along a supply chain leads to the bullwhip effect and thereby causes tremendous inefficiencies such as excessive inventory investment and lost revenue. The authors introduce four potential causes of the bullwhip effect, that is, demand forecast update, order batching, price fluctuations, and shortage gaming. Mason-Jones and Towill (2000) attributed the bullwhip effect to uncertainties in the supply side, manufacturing process, process control, and demand side of the supply chain. Meanwhile, numerous empirical, experimental, and analytical studies aim to find solutions to alleviate the bullwhip effect. There is a consensus in the literature that information sharing through collaboration among actors in the supply chain helps alleviate the bullwhip effect by reducing information distortion. Recently, Liu et al. (2022) observed that panic buying from human buyers during the lockdowns caused by COVID-19 leads to the bullwhip effect and can be mitigated by combining DRL algorithms and fictitious play.

For the measurement of the bullwhip effect, Wang and Disney (2016) provided an extensive review of various methods. We adopt a simple and effective measuring method introduced by Fransoo and Wouters (2000) in which the coefficient of variation is used to measure fluctuations in demand.

### 2.3. Multi-Agent Deep Reinforcement Learning

RL aims to find the optimal policy for MDPs by interacting with the environment and self-learning via collecting rewards. Previously proposed RL algorithms can be divided into three categories: value-based, policy-based, and actor-critic-based (Arulkumaran et al., 2017). In the value-based RL, a state-action value function $Q^{\pi }(s_{t},a_{t})$ is constructed to estimate the value of taking action $a_{t}$ in state $s_{t}$ under the current policy π . Along with iterations, $Q^{\pi }(s_{t},a_{t})$ is gradually adjusted to approximate the optimal action value function $Q*(s_{t},a_{t})$ and the best policy π* can be found by choosing $a_{t}$ optimally at every state $s_{t}:\max Q^{\pi }(s_{t},a_{t})$ . In the policy-based RL, rather than estimating a state-action value function, a parameterized policy function $\pi (s_{t})$ , which maps state $s_{t}$ to a deterministic action or action’s probability distribution, is constructed. Along with iterations, the policy function $\pi (s_{t})$ is adjusted towards the direction of maximizing the expected return, which is normally computed as a sum of discounted future rewards. As a hybrid of value-based and policy-based RL, the actor-critic-based RL includes both the policy function $\pi (s_{t})$ , namely the “actor,” and the value function $V^{\pi }(s_{t})$ , namely the “critic.” $V^{\pi }(s_{t})$ represents the value of being in state $s_{t}$ under policy π . Actor-critic-based RL uses the value function $V^{\pi }(s_{t})$ as a baseline to update the policy function $\pi (s_{t})$ .

Though with rigorous theoretical foundations, the successful applications of the above-mentioned RL algorithms are limited. One of the main reasons is that the function classes used to approximate the state-action function or policy function (i.e., tabular or linear function) are only feasible in low-dimensional problems. To address such a difficulty, DRL takes non-linear NNs as approximators and develops numerous well-performed algorithms by combining deep learning (DL) with RL. As a variant of a value-based RL algorithm called Q-learning, deep Q network (DQN) achieves tremendous success in various Atari games (Mnih et al., 2013). As one of the most classic actor-critic-based DRL algorithms, A3C is characterized by a novel asynchronous training scheme and achieves superior training efficiency and performance (Mnih et al., 2016). Based on the trust region learning proposed by Schulman et al. (2015), proximal policy optimization (PPO) outperforms the previous DRL algorithms by guaranteeing monotonical policy improvement in every training iteration. The superb performance and high scalability of the aforementioned DRL algorithms have inspired numerous applications in various domains, including inventory management. As mentioned, Gijsbrechts et al. (2021) applied A3C to three classic inventory problems and achieve desirable results. Oroojlooyjadid et al. (2022) constructed policies with DQN to play a decentralized beer game. Vanvuchelen et al. (2020) applied PPO to a joint replenishment inventory management problem and achieve much better performance than several heuristic policies.

Although DRL has become a promising method for solving practical problems, there still exists some complex real-world situations where the existing DRL methods may fail, an example of which is the MAS. In a MAS, multiple players cooperate or compete with each other to achieve the same or separate goals in a shared environment. To solve MDPs in MAS, DRL for MAS, that is, MADRL, has been developed. Among various existing MADRL algorithms, a direct extension of DRL to MADRL is to train each agent with a DRL algorithm while taking other agents as part of the environment. Such naive MADRL algorithms typically perform poorly because other agents’ actions would make the environment highly non-stationary, which creates difficulties for models’ convergence. To address such a limitation, a novel CTDE scheme for MADRL has been proposed. In CTDE, all agents are trained with full environment information, while they determine actions only based on their local information. Providing agents with more information makes the training easier, and executing with only local information makes MADRL more practical under situations where only partial observations are available or communication among agents is limited. By applying CTDE to PPO, Yu et al. (2021) introduced multi-agent PPO (MAPPO). They test MAPPO on three commonly used test environments for MADRL and show the superior performance of MAPPO on fully cooperative games. However, the application of MAPPO is limited since agents in MAPPO are required to be homogeneous. To address this issue, Kuba et al. (2021) proposed a sequential policy update scheme and introduce heterogeneous-agent PPO (HAPPO). Agents in HAPPO are heterogeneous, which is also the case in our problem, and the authors prove that the performance of HAPPO will be monotonically improved with policy updates.

## 3. MADRL in Serial Supply Chain System

In this section, we demonstrate how to apply MADRL to the multi-echelon inventory management problem in a serial supply chain system.

### 3.1. Model Formulation in Serial Supply Chain System

#### 3.1.1. Dynamics of Serial Supply Chain System

**Figure 3.**
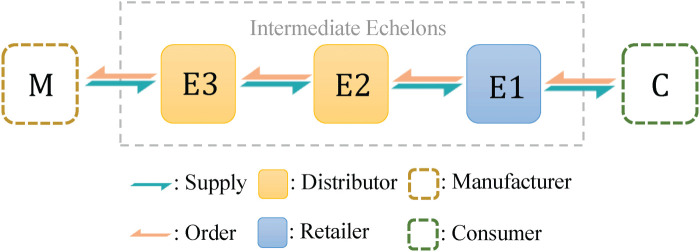
A serial supply chain system with three intermediate echelons.

We consider a periodic-review serial supply chain system containing M intermediate echelons between a manufacturer and customers. An example with M=3 is shown in Figure 3.

Since each echelon contains only one actor, we use integers from 1 to M to denote focal actors in intermediate echelons from downstream to upstream. To keep the notation consistent, we use integers 0 and M+1 to represent the customers and the manufacturer. Actor i∈{1,…,M} receives orders from actor i−1 and replenishes inventory from actor i+1 . As in the beer game, we assume that the inventory replenishment at any stage takes the same amount of a fixed lead time denoted by L . We use $I_{t}^{i}$ , $B_{t}^{i}$ , and $O_{t}^{i}$ to denote actor i ’s on-hand inventory at the beginning of period t , actor i ’s backlogs (accumulative unmet demand from actor i−1 ) at the beginning of period t , and actor i ’s ordering quantity at period t , respectively.^1^ We use $O_{t}^{0}$ to represent customer demand in period t . For each actor i , we consider backlog costs and holding costs whose coefficients are given by $b^{i}$ and $h^{i}$ , respectively.

In each period t , the quantity of goods that are shipped by actor i is represented by $P_{t}^{i}$ . We use $P_{t}^{M+1}$ to represent the quantity of goods shipped by the manufacturer to actor M in period t . All goods shipped by actor i will be in transit for L periods and added to actor i−1 ’s stocks in period t+L . Hence, the in-transit orders for actor i at the beginning of period t can be represented by the vector $\left(P_{t-1}^{i+1},P_{t-2}^{i+1},\ldots ,P_{t-L}^{i+1}\right)$ . For each period t , actor i ’s sellable goods are made up of its remaining inventory $I_{t}^{i}$ and just arrived orders $P_{t-L}^{i+1}$ . Since each actor only has one upstream source for goods replenishment, its ordering is constrained by the inventory level of its immediate upstream supplier. Specifically, for any actor i∈{1,…,M−1} , when it places orders of more than $I_{t}^{i+1}+P_{t-L}^{i+2}$ , only $I_{t}^{i+1}+P_{t-L}^{i+2}$ amount of goods can be provided and added to actor i ’s in-transit orders. We assume that the manufacturer has infinite stocks at any period, which makes the most upstream intermediate actor M ’s ordering unconstrained. The unfulfilled demand faced by actor i in period t consists of downstream actor i−1 ’s current orders $O_{t}^{i-1}$ and actor i ’s own backlogs $B_{t}^{i}$ . Hence, actor i ’s quantity of shipment $P_{t}^{i}$ in period t is

(1) $$P_{t}^{i}=\min\{B_{t}^{i}+O_{t}^{i-1},I_{t}^{i}+P_{t-L}^{i+1}\}.$$

Since the manufacturer always has enough stocks, its quantity of shipment is the same as the quantity of actor M ’s orders, which can be given by the following equation:

(2) $$P_{t}^{M+1}=O_{t}^{M}.$$

The update of the backlog $B_{t}^{i}$ and inventory $I_{t}^{i}$ for actor i can be written as follows:

(3) $$\begin{array}{rl} B_{t+1}^{i} & =B_{t}^{i}+O_{t}^{i-1}-P_{t}^{i}, \end{array}$$

(4) $$\begin{array}{rl} I_{t+1}^{i} & =I_{t}^{i}+P_{t-L}^{i+1}-P_{t}^{i}. \end{array}$$

In period t , actor i ’s holding costs and backlog costs are $h^{i}I_{t}^{i}$ and $b^{i}B_{t}^{i}$ . Hence, actor i ’s costs $C_{t}^{i}$ are given by the following equation:

(5) $$C_{t}^{i}=h^{i}I_{t}^{i}+b^{i}B_{t}^{i}.$$

The overall costs of the system in period t can be expressed by the following equation:

$$C_{t}^{\text{total}}=\sum_{i=1}^{M}C_{t}^{i}=\sum_{i=1}^{M}h^{i}I_{t}^{i}+b^{i}B_{t}^{i}.$$

We consider a fully cooperative supply chain. The optimization target is to find a policy that minimizes expected long-term overall costs, that is,

(6) $$\min E[\sum_{t=0}^{\infty }\gamma^{t}C_{t}^{\text{total}}],$$

where γ∈(0,1] is a discount factor.

#### 3.1.2. Formulation of POMG

To apply MADRL, we formulate the serial supply chain system as a POMG (Littman, 1994), which can be defined by a tuple $<M,S,A^{1},\ldots ,A^{M},O^{1},\ldots ,O^{M},T,R^{1},\ldots ,R^{M}>$ . Here, M is the number of actors, S is the set of global states of the system, $A^{i}$ is the set of actions for actor i , $O^{i}$ is the set of observations for actor i , $T:S\times A^{1}\times \cdots \times A^{M}\times S\to [0,1]$ is the global state transition function that maps the current global state, all actors’ actions, and the next global state to a transition probability, and $R^{i}:S\times A^{i}\to R$ is the reward function for actor i . In each period t , every actor i receives a partial observation $o_{t}^{i}\in O^{i}$ under global state $s_{t}\in S$ and takes a randomized action $a_{t}^{i}∼\pi^{i}(\cdot |o_{t}^{i})$ , where $\pi^{i}$ is a policy which maps the observation to action’s probability distribution. After all actors have taken their actions, each actor i receives a reward $R^{i}(a_{t}^{i}|s_{t})$ , and the system transits to the next global state $s_{t+1}∼T(\cdot |a_{t}^{1},\ldots ,a_{t}^{M},s_{t})$ .

Following the above scheme, we treat actor i ’s ordering quantity $O_{t}^{i}$ as actor i ’s action $a_{t}^{i}$ and develop a discrete action space for each actor as $A^{i}=\{0,\ldots ,O^{\max}\}$ , where $O^{\max}$ denotes the maximum ordering quantity. To make sure that an observation contains enough information, including the on-hand inventory, backlogs, downstream demand, and in-transit orders, we define actor i ’s partial observation $o_{t}^{i}\in O^{i}$ as

(7) $$o_{t}^{i}=(I_{t}^{i},B_{t}^{i},O_{t-1}^{i-1},P_{t-L}^{i+1},\ldots ,P_{t-1}^{i+1}).$$

For the reward function, we define actor i ’s reward as follows:

(8) $$r_{t}^{i}=R^{i}(a_{t}^{i}|s_{t})=-\alpha C_{t}^{i}-(1-\alpha )C_{t}^{\text{total}}.$$

Here α∈[0,1] is an exogenous weight that measures how self-interested the actor is during decision-making. When α=0 , each actor considers the overall costs of a supply chain. When α=1 , each actor only focuses on reducing its own costs. The introduction of parameter α in the actor’s reward seems unnatural at first glance. With the objective (6) of minimizing the overall costs of the system, the reward for each actor should be intuitively set as the opposite of overall costs, which is the case of α=0 . However, under the context of MADRL, the parameter α affects the performance of trained actors through a unique bias-variance trade-off. To see this, we establish the following Propositions 1 and 2, which demonstrate the bias aspect and the variance aspect of the trade-off, respectively. The proofs of Propositions 1 and 2 are given in Appendix A.

Proposition 1 When applying a joint policy $\left(\pi^{1},\ldots ,\pi^{M}\right)$ , the bias $|E[\sum_{t=0}^{\infty }\gamma^{t}r_{t}^{i}]-E[-\sum_{t=0}^{\infty }\gamma^{t}C_{t}^{\text{total}}]|$ for any actor i∈{1,…,M} is monotone increasing w.r.t. the parameter α and is 0 with α=0 .

When applying MADRL algorithms, we need to evaluate the current joint policy $\left(\pi^{1},\ldots ,\pi^{M}\right)$ before conducting each policy update. Specifically, we simulate the decision processes for a certain number of episodes with the current joint policy and record the simulated rewards as the estimation of the expected rewards $E[\sum_{t=0}^{\infty }\gamma^{t}r_{t}^{i}]$ for actor i . The policy of each actor i is then updated towards the direction of increasing the simulated rewards. With the objective of minimizing the overall costs of the system, the expected rewards $E[\sum_{t=0}^{\infty }\gamma^{t}r_{t}^{i}]$ should be close to the opposite of the expected overall costs $E[\sum_{t=0}^{\infty }\gamma^{t}C_{t}^{\text{total}}]$ such that the policy update of actor i simultaneously leads to the decrease of the expected overall costs. Proposition 1 shows that as α decreases to 0, the bias $|E[\sum_{t=0}^{\infty }\gamma^{t}r_{t}^{i}]-E[-\sum_{t=0}^{\infty }\gamma^{t}C_{t}^{\text{total}}]|$ also decreases to 0. In other words, with a smaller α , increasing the expected rewards decreases the expected overall costs of the system more significantly.

Proposition 2 When applying a joint policy $\left(\pi^{1},\ldots ,\pi^{M}\right)$ , for any actor i∈{1,…,M} , if it holds that $\text{Cov}(\sum_{t=0}^{\infty }\gamma^{t}C_{t}^{i},\sum_{t=0}^{\infty }\sum_{j\in \{1,\ldots ,M\}/\{i\}}\gamma^{t}C_{t}^{j})\geq 0$ , the variance $\text{Var}(\sum_{t=0}^{\infty }\gamma^{t}r_{t}^{i})$ of accumulated rewards for actor i is monotone decreasing w.r.t. the parameter α .

Proposition 2 shows that as α decreases, the variance of the accumulated reward $\sum_{t=0}^{\infty }\gamma^{t}r_{t}^{i}$ for any actor i increases under the condition that costs of actor i have a positive correlation with total costs of other actors. This means that when α decreases, the estimation of the expected reward $E[\sum_{t=0}^{\infty }\gamma^{t}r_{t}^{i}]$ is less accurate with the same number of simulation episodes.

Propositions 1 and 2 thereby form a bias-variance trade-off controlled by the parameter α that affects the performance of the MADRL algorithms. Specifically, consider a certain iteration where we aim to update the current joint policy $\left(\pi^{1},\ldots ,\pi^{M}\right)$ . On the one hand, larger α makes the policy evaluation more accurate and thereby improves the policy towards the optimization target more effectively. On the other hand, larger α makes the optimization target deviate more from the true common objective. Hence, the performance of the updated policy after this iteration is affected by the trade-off controlled by the parameter α . After a sequence of policy iterations, the performance of the eventually obtained joint policy is affected by the parameter α in a similar manner. In our considered supply chain setting, the experimental results in Section 3.4 show that the best-performing α is attained at a certain value between 0 and 1 , which confirms the aforementioned argument to a large extent.

In addition, the introduced bias-variance trade-off has three distinctive features. First, it is unique to MADRL since the variance aspect is essentially caused by the fact that MADRL, as a numerical method, uses finite simulations to estimate the expected reward. Second, it is unique to the cooperative multi-echelon inventory setting where each actor has a positive linear contribution, for example, individual costs, to the ultimate common objective, for example, overall costs of all actors. Third, it is unique to the reward definition (8), which is a convex combination of the individual contribution of each actor and the ultimate common objective of the system. Therefore, when applying MADRL on cooperative multi-echelon inventory systems with an additively contributed objective, for example, the overall costs across echelons, the aforementioned trade-off implies that setting the individual objective for each actor as the common objective may not give the best performance because of the practical training mechanism of MADRL. One can consider applying the reward shaping (8) and conduct additional experiments to find the optimal α , which reflects the optimal degree for actors to be self-interested. This also provides valuable managerial insights that when applying MADRL to multi-echelon inventory systems, one needs to consider both the managerial target and the effectiveness of MADRL algorithms to determine the optimization objective for each actor.

#### 3.1.3. Generation of Stochastic Customer Demand

To simulate a volatile environment where the bullwhip effect is more likely to happen, we generate non-stationary customer demand by a Merton jump diffusion model (MJD) (Merton, 1976). Originally used to simulate stock price data with high peaks and heavy tails, MJD adds jumps to a diffusion process to model sudden changes. Such features can well capture customer demands in the real world where bursts or stagnant sales randomly appear due to external shocks such as the COVID-19 outbreaks. We simulate MJD by a method by Glasserman (2004). Specifically, we compute the demand $O_{t}^{0}$ and the t -th element $J_{t}$ in a MJD sequence as follows:

(9) $$\begin{array}{rl} O_{t}^{0} & =\left\lfloor \ell_{1}\exp\;(J_{t})\right\rfloor ,\\ J_{t} & =J_{t-1}+\left(\mu -\frac{1}{2}\sigma^{2}\right)+\sigma Z_{t}+\ell_{2}N_{t}+\ell_{3}\sqrt{N_{t}}Z_{t}^{\prime }, \end{array}$$

where $Z_{t}$ , $N_{t}$ , and $Z_{t}^{\prime }$ are i.i.d random variables, and $Z_{t}∼\text{Normal}(0,1)$ , $Z_{t}^{\prime }∼\text{Normal}(0,1)$ , $N_{t}∼\text{Poisson}(\lambda )$ . Values for the six exogenous parameters μ , σ , λ , $\ell_{1}$ , $\ell_{2}$ , and $\ell_{3}$ are provided in Appendix G.

**Figure 4.**
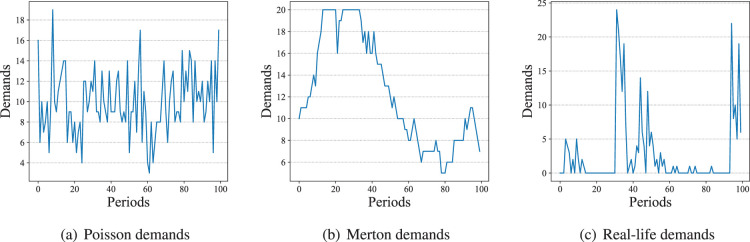
Examples of Poisson, Merton, and real-life demands.

Other than the Merton demands, we also conduct some experiments with Poisson distributed demands and real-life demands. Provided in the M5 forecasting competition (Howard et al., 2020), the real-life demand data comes from the retailing goods of Walmart. The statistical information of the real-life data we use is summarized in Appendix B. Examples of Merton, Poisson, and real-life demands are provided in Figure 4.

### 3.2. MADRL Model Construction

**Figure 5.**
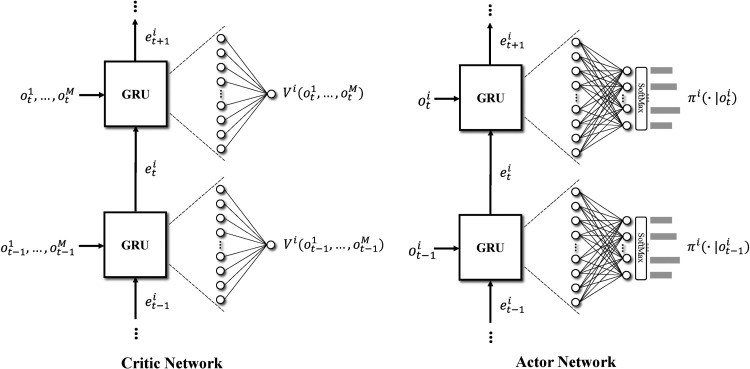
Diagram of actor and critic networks based on RNN with GRU. RNN: recurrent neural network; GRU: gate recurrent unit.

We apply HAPPO to construct ordering policies for the POMG defined in Section 3.1. HAPPO applies an on-policy PPO for each actor and follows a CTDE structure. For every actor i , a policy function $\pi^{i}$ , which maps i ’s partial observation to the probability distribution of action $\pi^{i}(\cdot |o_{t}^{i})$ , and a value function $V^{i}$ , which maps all actors’ observations to state value $V^{i}(o_{t}^{1},\ldots ,o_{t}^{M})$ of actor i , are constructed and parameterized by NNs. The policy function and the value function are also known as the actor network and the critic network. To construct actor and critic networks, previous papers such as Gijsbrechts et al. (2021) and Oroojlooyjadid et al. (2022) use simple fully connected networks to construct their models. Such neglect of historical information, including demand fluctuations and changes in inventory levels, may lead to inferior decisions of DRL algorithms. To address the limitation, we choose a recurrent NN (RNN) with a gate recurrent unit (GRU) to construct our actor and critic networks. As an extension of conventional feedforward NNs, the RNN can learn temporal dependencies among input data by maintaining a hidden state that contains the information of historical inputs. Some sophisticated recurrent units are introduced in the RNN to capture better dependencies of different time scales, one of which is the GRU (see Chung et al. 2014 for more details about the RNN and the GRU). We illustrate the diagram for actor and critic networks developed with the RNN and the GRU in Figure 5.

For the actor network, in each period t , a GRU receives the current observation $o_{t}^{i}$ and a hidden state $e_{t}^{i}$ as inputs and outputs an updated hidden state $e_{t+1}^{i}$ and some high-dimensional features. These features are then down-sampled to match the size of the action space with a fully connected layer and mapped into the probability distribution of actions by a softmax layer. The only difference between the critic network and the actor network is that features produced by GRU are mapped into the state value instead of the probability distribution of actions.

For the actual training process of each actor i , we use $\pi_{k}^{i}$ and $V_{k}^{i}$ to represent actor and critic networks after k updates. For each episode, we simulate the POMG associated with the serial supply chain system for finite T periods by applying the current policy ( $\pi_{k}^{1},\ldots ,\pi_{k}^{M}$ ) and collect all actors’ observations, actions, and rewards as the training data. Then, the actor network $\pi_{k}^{i}$ and the critic network $V_{k}^{i}$ are updated to $\pi_{k+1}^{i}$ and $V_{k+1}^{i}$ with the collected data for each actor i . A pseudocode for the detailed simulation and parameter update process is presented in Appendix C. For the fully cooperative POMG formulated with α=0 , the expected overall costs of the supply chain are guaranteed to be improved monotonically by the sequential policy update scheme in HAPPO, which is shown in the following Proposition 3.

Proposition 3 By applying HAPPO to the POMG constructed from the serial supply chain with α=0 , the expected overall costs of the supply chain decrease monotonically with policy updates, that is,

$$\begin{array}{rl} & E_{a_{0:\infty }^{1}∼\pi_{k+1}^{1},\ldots ,a_{0:\infty }^{M}∼\pi_{k+1}^{M},s_{0:\infty }∼T}\left[\sum_{t=0}^{\infty }\gamma^{t}C_{t}^{\text{total}}\right]\\ & \;\leq E_{a_{0:\infty }^{1}∼\pi_{k}^{1},\ldots ,a_{0:\infty }^{M}∼\pi_{k}^{M},s_{0:\infty }∼T}\left[\sum_{t=0}^{\infty }\gamma^{t}C_{t}^{\text{total}}\right], \end{array}$$

where $a_{0:\infty }^{i}∼\pi_{k}^{i}$ represents that all actions during the decision process are made according to the policy $\pi_{k}^{i}$ .

The proof of Proposition 3 can be found in Appendix A.

### 3.3. Baseline Policies and Experiment Settings

We implement both heuristic and DRL policies as baselines to evaluate HAPPO’s performance. Since most of our experiments are conducted using non-stationary Merton and real-life demands, we implement non-stationary base-stock and ( s , S ) policies as heuristic baselines. Specifically, we adopt the non-stationary base stock policy introduced by Neale and Willems (2009) and the non-stationary ( s , S ) policy introduced by Bollapragada and Morton (1999). The implementation details of non-stationary base stock and non-stationary ( s , S ) policies are provided in Appendix D. For DRL policies, we adopt PPO separately for each actor to minimize its own costs and train each actor with the same setting as used in HAPPO, including the training demand data, the NN structure, and hyperparameters. Note that two major differences between HAPPO and the PPO baseline are (1) HAPPO takes a CTDE scheme where information is shared during training, while each actor in the PPO baseline is trained only based on its local information; (2) in HAPPO, each actor considers both its own costs and the overall costs of the system, whereas each actor in the PPO baseline only minimizes its own costs. Hence, the difference in performance between HAPPO and the PPO baseline can be mainly attributed to information sharing and cooperation inside the supply chain during the training process.

We design the following six sets of experiments to evaluate the performance of HAPPO.

#### 3.3.1. Optimality Test in Serial Supply Chain System

To validate HAPPO’s convergence, we consider two simplified problems in the serial supply chain system where the optimal policy is tractable.

First, we consider the deterministic setting of zero lead time, no startup inventory, no startup backlog, and deterministic demand, which means the external demand $O_{t}^{0}$ is deterministic and known for any period t≥0 . We show by Proposition 4 that the optimal policy that minimizes the overall costs of the supply chain under this scenario is to order the same amount of goods as the deterministic customer demand for every actor in each period. The proof of Proposition 4 is provided in Appendix A. We apply HAPPO to this simplified serial supply chain system and test whether it can converge to the optimal policy.

Proposition 4 For the serial supply chain system with M echelons, if the following conditions are met: (i) zero lead time L=0 , (ii) no startup inventory $I_{0}^{i}=0,\;\forall i\in \{1,\ldots ,M\}$ , and (iii) no startup backlog $B_{0}^{i}=0,\;\forall i\in \{1,\ldots ,M\}$ , the optimal ordering policy that minimizes the overall long-term costs of the supply chain can be given by the following equation:

$$O_{t}^{i}=O_{t}^{0},\;\forall i\in \{1,\ldots ,M\},\;t\geq 0,$$

where $O_{t}^{0}$ represents the customer demand in period t .

Then, we consider the stochastic setting of non-zero lead time, no startup inventory, no startup backlog, and stochastic demands. The demand $O_{t}^{0}$ for any period t≥0 is an i.i.d. Poisson distributed random variable. What’s more, the backlog costs only occur at the most downstream actor, that is, $b^{1}=1$ and $b^{i}=0$ for any i∈{2,…,M} . Clark and Scarf (1960) established the optimality of the base stock policy in a serial supply chain system under the aforementioned setting. Shang and Song (2003) further provided a heuristic method to find the optimal base stock levels. We compute the optimal base stock levels by using the method by Shang and Song (2003), whose details are provided in Appendix E, and test whether HAPPO can converge to the optimal base stock policy.

#### 3.3.2. Effect of α on the Performance of HAPPO

As mentioned, parameter α in the reward function (8) controls how self-interested the actor is during decision-making. To test the effect of α on the performance of HAPPO, we conduct controlled experiments on α by training HAPPO models with different values of α and comparing the average overall costs achieved on the Merton demand data.

#### 3.3.3. Policy Comparison in a Typical Serial Supply Chain System

We evaluate the performance of HAPPO and other baseline policies in a typical serial supply chain system with three, four, and five echelons by using Merton and real-life demands, respectively. We use the overall costs as our metrics for the numerical comparison of different policies. To evaluate the bullwhip effect, as introduced in Section 2.2, we use the coefficient of variation as our metric. Specifically, for actor i , we measure its demand fluctuation during the finite simulation periods {0,…,T} as follows: $\sqrt{\sum_{t=0}^{T}{\left(O_{t}^{i-1}-{\bar{O}}^{i-1}\right)}^{2}}/(\sqrt{T+1}\;{\bar{O}}^{i-1}),$ where ${\bar{O}}^{i-1}=\frac{1}{T+1}\sum_{t=0}^{T}O_{t}^{i-1}$ . Other than the problem setting introduced in Section 3.1, we also conduct experiments on the serial supply chain system with fixed costs. We model it into the actor’s cost structure as follows:

$$C_{t}^{i}=h^{i}I_{t}^{i}+b^{i}B_{t}^{i}+f^{i}1(P_{t}^{i+1}>0),$$

where $f^{i}$ is the coefficient of fixed cost for actor i and $1(P_{t}^{i+1}>0)$ is an indicator function that takes the value of 1 when actor i ’s upstream actor i+1 ships goods to actor i in period t , that is, $P_{t}^{i+1}>0$ , and takes the value of 0 otherwise.

#### 3.3.4. Sensitivity Analysis With Different Lead Times and Cost Coefficients

To verify the robustness of policies constructed by HAPPO, we evaluate HAPPO and baseline policies’ sensitivity with respect to different lead times and different cost coefficients when using Merton demands without fixed costs. Since lead time determines the size of the observation space (see Section 3.1), we train different HAPPO and PPO models for different lead times. For the sensitivity to cost coefficients, we train those models under one set of cost coefficients and evaluate their performances under other settings of cost coefficients.

#### 3.3.5. Policy Comparison With Different Information Sharing Conditions

As mentioned in Section 3.2, a typical MADRL method using CTDE assumes that each actor has access to all other actors’ information during training and only has access to its local information during execution. This set of experiments aims to study whether decentralization in execution harms the performance of HAPPO when applied to inventory management. We construct a baseline called “centralized HAPPO,” where each actor has access to all other actors’ information not only during training but also during execution. More precisely, the input to the actor network $\pi^{i}$ for any actor i is the full observation $\left(o_{t}^{1},\ldots ,o_{t}^{M}\right)$ instead of simply its local observation $o_{t}^{i}$ . Intuitively, the centralized HAPPO should outperform HAPPO since it has access to more information.

Besides, under certain scenarios of supply chain management, one actor may only have access to information about its neighbors. Practical evidence also shows that the limited observations can be an important reason for the sudden collapse of modern “just in time” supply chains (Dvorak, 2009). Hence, to test HAPPO’s performance with the limited observations during training, we change the input of value network $V^{i}$ for actor i in HAPPO from full observations $\left(o_{t}^{1},\ldots ,o_{t}^{M}\right)$ to limited observations $\left(o_{t}^{i-1},o_{t}^{i},o_{t}^{i+1}\right)$ where only information of actor i ’s direct supplier i+1 and direct customer i−1 is available. The experiments are conducted by using Merton demands without fixed costs.

#### 3.3.6. Policy Comparison With Price Discounts and Random Shipping Loss

As introduced by Lee et al. (1997), price discounts provided by upstream actors can aggravate the bullwhip effect because it leads to bulk purchases from downstream actors and thereby causes the distortion of real demand information. To examine the bullwhip effect when the ordering decision is made by actors trained by HAPPO in the presence of price discounts, we change the cost structure in equation (5) by introducing ordering costs. We use $c^{i}$ to represent the base per-unit ordering costs for actor i and introduce a discount factor to simulate the price discounts provided by suppliers. Actor i ’s costs under this scenario are given by the following equation:

$$C_{t}^{i}=h^{i}I_{t}^{i}+b^{i}B_{t}^{i}+c^{i}F(\frac{O_{t}^{i}}{O^{\max}})O_{t}^{i},$$

where the discount factor F:[0,1]→[0,1] is a monotonely decreasing step function whose detailed values are shown in Appendix G.

Other than price discounts, uncertainties in multiple stages of the supply chain also contribute to the bullwhip effect (Mason-Jones and Towill, 2000). Hence, we create additional uncertainties in the system by introducing a random shipping loss. Suppose a random portion of goods is spoiled during the shipment. The quantity of shipment in equation (1) and inventory update in equation (4) under this scenario can be modified as follows:

(10) $$\begin{array}{rl} P_{t}^{i} & =\min\{B_{t}^{i}+O_{t}^{i-1},I_{t}^{i}+\lfloor \delta^{i}P_{t-L}^{i+1}\rfloor \},\\ I_{t+1}^{i} & =I_{t}^{i}+\lfloor \delta^{i}P_{t-L}^{i+1}\rfloor -P_{t}^{i}, \end{array}$$

where the shipping loss factor $\delta^{i}$ is a random variable sampled from a uniform distribution. The experiments are conducted using Merton demands without fixed costs.

Exogenous parameters used in the aforementioned experiments, including cost coefficients and lead times, are summarized in Table 1. For all policies, we conduct the evaluation on 20 different demand traces and record the average value of the considered metrics on 20 demand traces. For each experiment conducted on HAPPO and the PPO baseline, we repeat the training processes for 10 times to reduce the randomness caused by the model’s training. The mean and the confidence interval of considered metrics that come from 10 repeated training processes are presented in related figures. For heuristic policies, the evaluation only needs to be conducted for one time, and the average value of metrics on 20 demand traces is presented in related figures.

**Table 1.** Coefficients in experiments conducted on the serial supply chain system.

	Experiments					
Coefficients	Effect of α	Typical	Sensitivity analysis	Information conditions	Price discounts	Random shipping loss
Lead time L	{2,4}	4	{1,2,4,6}	4	4	4
Holding costs $h^{i}$	{1,0.5,0.3}	1	{0.5,0.55,…,1.5}	1	1	1
Backlog costs $b^{i}$	1	1	{0.5,0.55,…,1.5}	1	1	1
Ordering costs $c^{i}$	–	–	–	–	1	–
Fixed costs $f^{i}$	–	5	–	–	–	–
Shipping loss Factor $\delta^{i}$	–	–	–	–	–	Uniform(0.9,1)

Note. For each experiment above, all actors in the system use the same setting of coefficients. “–” means “not applicable.”

### 3.4. Numerical Results

In this section, we provide numerical results and related discussions for experiments in Section 3.3.

#### 3.4.1. Results of Optimality Test in a Simplified Serial Supply Chain System

**Figure 6.**
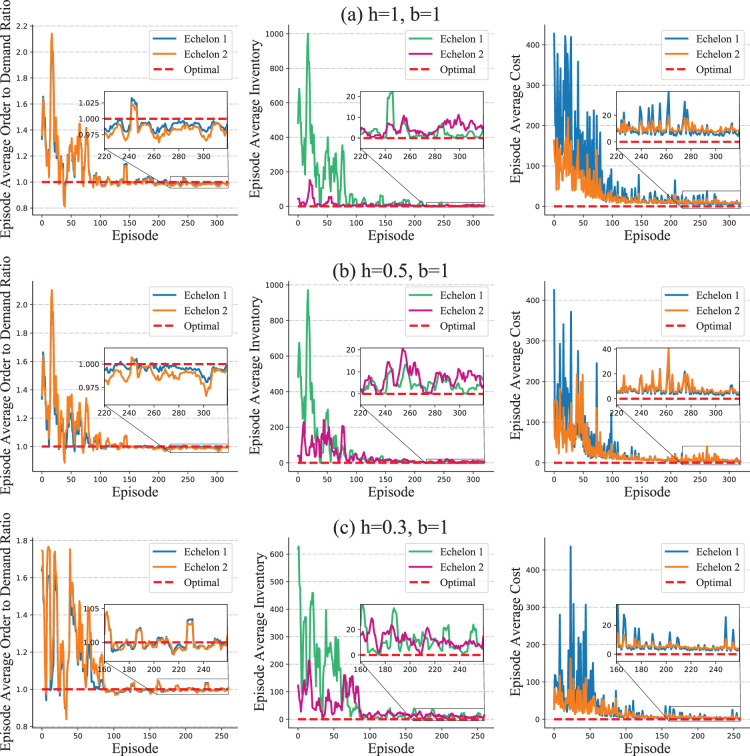
Optimality test in simplified serial supply chain system with deterministic demands.

**Figure 7.**
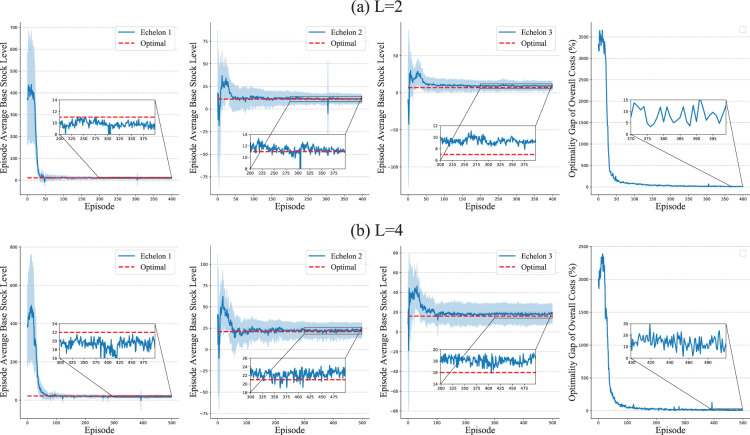
Optimality test in simplified serial supply chain system with stochastic demands.

For the deterministic setting, we consider a two-echelon system and record each echelon’s average ordering, average inventory, and average costs for every training episode. We consider three different settings of coefficients for holding and backlog costs, that is, $h^{i}=b^{i}$ , $h^{i}=0.5b^{i}$ , and $h^{i}=0.3b^{i}$ . The results are presented in Figure 6. Although HAPPO, as a numerical method, does not converge strictly to the optimal values of the order-to-demand ratio, inventory, and costs, the difference between the optimal values and results of HAPPO significantly reduces during training. For the stochastic setting, we consider a three-echelon system. We record each echelon’s average base stock level, which is the average value of on-hand inventory plus outstanding orders minus backlogs for each training episode, and the overall costs of the system. We consider two settings with different lead times, that is, L=2 and L=4 . The results in Figure 7 show that the base stock levels achieved by HAPPO gradually stabilize along with the training. The difference between the base stock levels achieved by HAPPO and the optimal values significantly reduces during the training. The optimality gap of HAPPO in terms of the overall costs reduces from more than 1500% to around 15% after the training. Hence, under both the deterministic and the stochastic settings, HAPPO almost closes the gap with the known optimal policies, which indicates the effectiveness of HAPPO for multi-echelon inventory problems.

#### 3.4.2. Effect of α on the Performance of HAPPO

**Figure 8.**
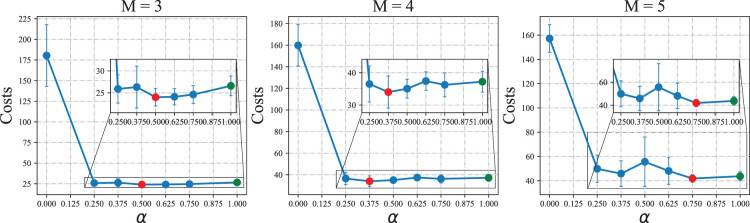
Effect of parameter α on heterogeneous-agent proximal policy optimization (HAPPO) model’s performance in the serial supply chain. Note. The red point in each figure represents the lowest overall costs achieved by α between 0 and 1, and the green point in each figure represents the overall costs achieved by α=1 .

We present the overall costs of the system associated with different values of α in Figure 8 and make the following intriguing observation 1.

Observation 1 *When applying HAPPO on a serial supply chain, the best performing α that achieves the lowest overall costs of the system is between 0 and 1, that is, each actor considers minimizing a combination of its individual costs and overall costs of the system.*

Observation 1 shows that HAPPO achieves the best practical performance when each actor considers both its own costs and other actors’ costs. This phenomenon is consistent with our analysis in Section 3.1. The parameter α controls a bias-variance trade-off, which explains the bad performance of the fully cooperative objective, that is, α=0 , from the perspective of the MADRL algorithm. According to the results, we determine the values of α for other experiments as the ones that yield the lowest overall costs, that is, α=0.5 for the system with three echelons, α=0.375 for the system with four echelons, and α=0.75 for the system with five echelons.

#### 3.4.3. Results of Policy Comparison in a Typical Serial Supply Chain System

**Figure 9.**
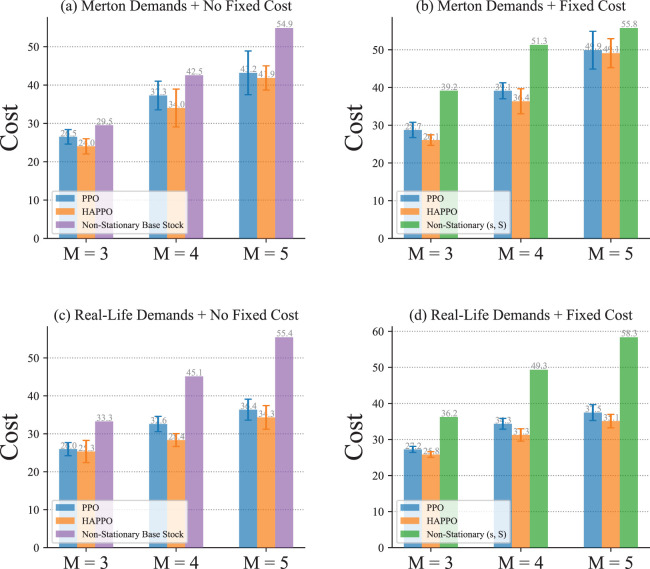
Comparison of system’s overall costs in a typical serial supply chain.

With non-stationary external demands, results in Figure 9 show that HAPPO achieves the lowest overall costs, and both HAPPO and the PPO baseline outperform the non-stationary base stock and ( s , S ) policies under all settings. These results demonstrate that with minimal information sharing needed only in the training stage, HAPPO achieves significantly better performance than the other baseline policies with no information sharing.

**Figure 10.**
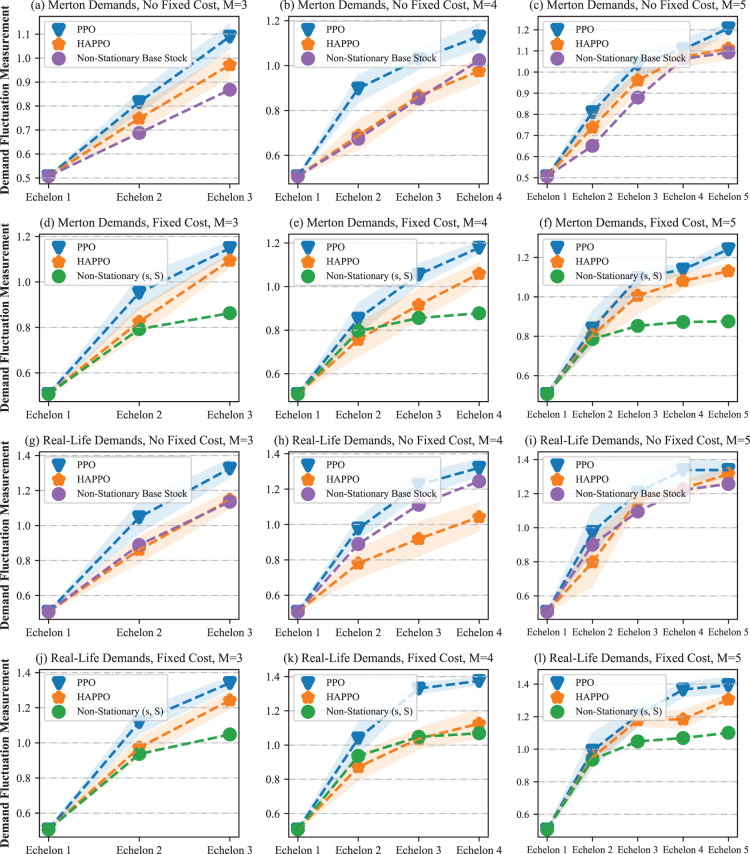
Comparison of the bullwhip effect in a typical serial supply chain.

The comparison of the bullwhip effect in the serial supply chain system is shown in Figure 10. The bullwhip effect that demand fluctuation amplifies from downstream to upstream is more significant in the PPO baseline compared to HAPPO. Specifically, as shown in Table 2, both the average and the amplification of demand fluctuation are smaller in HAPPO compared to the PPO baseline, which implies that the bullwhip effect is effectively alleviated by HAPPO. These results indicate the effectiveness of the upfront-only information-sharing mechanism in HAPPO and also provide an explanation for HAPPO’s superiority in reducing the overall costs of the system. We provide a policy visualization in the serial supply chain system in Appendix I.

#### 3.4.4. Sensitivity Analysis With Different Lead Times and Cost Coefficients

**Figure 11.**
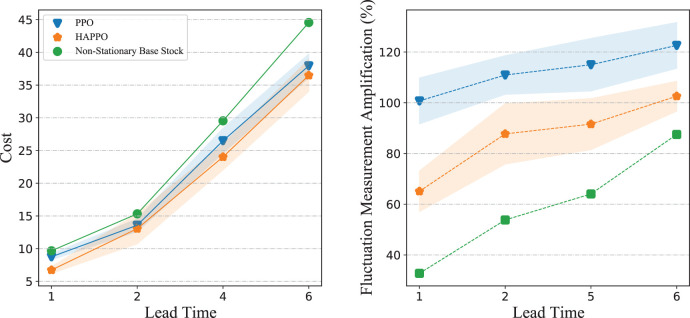
Sensitivity of different policies with respect to different lead times.

We conduct sensitivity analysis on a typical serial supply chain system with three echelons. As shown in Figure 11, HAPPO constantly achieves the lowest overall costs with different lead times, which demonstrates HAPPO’s robustness to changes in lead time. The demand fluctuation amplification for both the PPO baseline and HAPPO is generally larger when lead time increases, which is consistent with the argument by Lee et al. (1997) that a longer lead time aggravates the bullwhip effect. Nevertheless, the demand fluctuation amplification for HAPPO is smaller than that for the PPO baseline in all settings, which demonstrates HAPPO’s robustness to changes in lead time in terms of alleviating the bullwhip effect.

**Figure 12.**
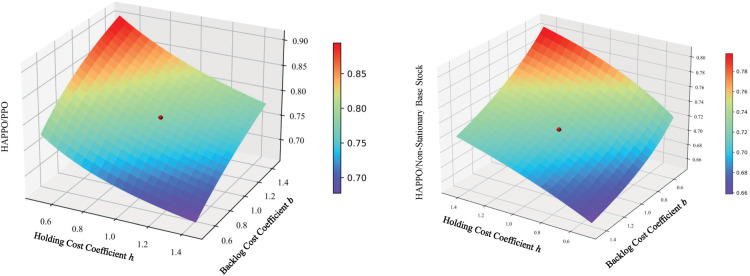
Sensitivity of different policies with respect to different cost coefficients. Note. The *Z*-axis is the cost ratio of heterogeneous-agent proximal policy optimization (HAPPO) to different baseline policies. The red point in each figure represents the settings of cost coefficients used in training.

For policies’ sensitivity to cost coefficients, Figure 12 shows that HAPPO achieves the lowest overall costs in all settings we consider. The results also show that HAPPO still maintains its superiority under the parameter settings that are not used during training. This indicates the desirable generalization ability of policies constructed by HAPPO.

#### 3.4.5. Results of Policy Comparison With Different Information Sharing Conditions

**Figure 13.**
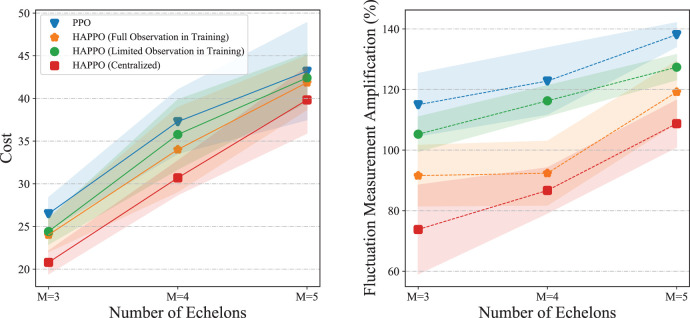
Comparison of system’s overall costs and bullwhip effect under different information-sharing conditions.

We conduct the comparison of overall costs and bullwhip effect with different information-sharing conditions. From the results shown in Figure 13, we summarize the following Observation 2.

Observation 2 *When applying HAPPO and PPO on a serial supply chain, the overall costs of the supply chain system decrease, and the bullwhip effect alleviates as the information sharing increases in the order of PPO → HAPPO with limited observation during training → HAPPO with full observation during training → centralized HAPPO.*

Observation 2 firstly indicates that more information sharing leads to better performance of actors constructed with DRL algorithms. Also, Observation 2 is consistent with the intuition that more information sharing leads to a less significant bullwhip effect in a serial supply chain. What’s more, HAPPO is still effective in alleviating the bullwhip effect even when each actor only has access to information about its two neighbors in the supply chain during the training. What should be motioned is that although the centralized HAPPO shows superiority over the other considered methods, it needs the “central control tower” assumption introduced before and thereby may be unimplementable in the practice of multi-echelon supply chain management.

We also conduct a comparison of the convergence speeds for HAPPO with different information-sharing conditions. The results of training episodes needed to achieve the best performance for different methods are presented in Table 3. In general, centralized HAPPO converges faster than HAPPO with full observation during training, which converges faster than HAPPO with limited observation during training. These results indicate that more information sharing leads to a faster convergence speed. An intuitive explanation for such a phenomenon is that with more information provided, it is easier for each actor to capture the dynamics of the whole inventory system and thereby faster to converge to a desirable policy.

**Table 2.** Statistics of demand fluctuation under different experiment settings.

	Demand fluctuation measurement	
	(HAPPO, PPO, non-stationary ( s , S ), non-stationary base stock)	
Experiment settings	Average	Amplification (%)
Merton demands, no fixed cost, *M* = 3	(0.742, 0.804, –, 0.687)	(91.51, 114.9, –, 71.26)
Merton demands, no fixed cost, *M* = 4	(0.758, 0.890, –, 0.764)	(92.37, 122.8, –, 101.9)
Merton demands, no fixed cost, *M* = 5	(0.877, 0.933, –, 0.838)	(119.1, 138.1, –, 115.6)
Merton demands, fixed cost, *M* = 3	(0.808, 0.869, 0.720, –)	(115.7, 126.5, 70.14, –)
Merton demands, fixed cost, *M* = 4	(0.809, 0.898, 0.758, –)	(108.8, 132.4, 73.09, –)
Merton demands, fixed cost, *M* = 5	(0.905, 0.964, 0.778, –)	(123.0, 144.8, 72.65, –)
Real-life demands, no Fixed cost, *M* = 3	(0.838, 0.960, –, 0.843)	(126.3, 161.6, –, 124.0)
Real-life demands, no fixed cost, *M* = 4	(0.811, 1.007, –, 0.938)	(105.5, 160.4, –, 145.4)
Real-life demands, no fixed cost, *M* = 5	(1.002, 1.072, –, 0.996)	(126.3, 161.6, –, 124.0)
Real-life demands, fixed cost, *M* = 3	(0.907, 0.988, 0.830, –)	(144.9, 164.4, 106.6, –)
Real-life demands, fixed cost, *M* = 4	(0.885, 1.062, 0.890, –)	(121.9, 171.0, 110.8, –)
Real-life demands, fixed cost, *M* = 5	(1.025, 1.093, 0.932, –)	(157.6, 175.0, 117.1, –)

Note. Each value in the “Average” column is computed as $\frac{1}{M}\sum_{i=1}^{M}F^{i}$ and each value in the “Amplification (%)” column is computed as $\frac{F^{M}}{F^{1}}-1$ , where $F^{i}$ denotes the demand fluctuation measurement for actor i . HAPPO = heterogeneous-agent proximal policy optimization; PPO = proximal policy optimization.

#### 3.4.6. Results of Policy Comparison With Price Discounts and Random Shipping Loss

**Figure 14.**
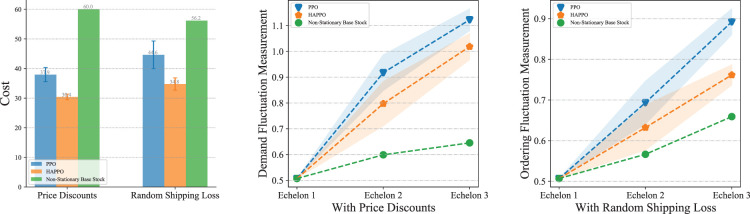
Comparison of system’s overall costs and bullwhip effect with price discounts and random shipping loss.

The experiments are conducted on the serial system with three echelons. The comparison of overall costs in Figure 14 shows that HAPPO still achieves the lowest overall costs with both the price discounts and the random shipping loss. Also, Figure 14 shows that the bullwhip effect is less significant by using HAPPO than the PPO baseline in both two settings. Table 4 shows that the demand fluctuation amplifies by 121% in the PPO baseline with the price discounts (114% without price discounts shown in Table 2) and 100% in HAPPO with the price discounts (91% without price discounts shown in Table 2) along the supply chain. These results substantiate that adding price discounts to the system aggravates the bullwhip effect for both the PPO baseline and HAPPO, which is consistent with the empirical finding by Lee et al. (1997) that suppliers providing price discounts is an important cause for the bullwhip effect. In contrast, the random shipping loss contributes to a surprising decrease in demand fluctuation amplification for both HAPPO and the PPO baseline, that is, a decrease from 91% (Table 2) to 50% for HAPPO and a decrease from 114% (Table 2) to 75% in the PPO baseline. An explanation is that the random shipping loss brings uncertainties into the actual quantities of goods actors receive in each period. Hence, instead of making orders by closely following the fluctuations of customer demand, actors learn to order a bit more and steadily to deal with such uncertainties in goods replenishment.

**Table 3.** Comparison of training episodes for heterogeneous-agent proximal policy optimization (HAPPO) under different information-sharing conditions.

	Methods		
	HAPPO	HAPPO	HAPPO
echelons	(centralized)	(full observation)	(limited observation)
*M* = 3	260 ± 33	299 ± 59	311 ± 47
*M* = 4	275 ± 51	327 ± 35	375 ± 41
*M* = 5	289 ± 33	349 ± 36	376 ± 35

Note. The training episodes are provided with the mean and 95% confidence interval of 10 repeated training processes.

**Table 4.** Statistics of demand fluctuation with price discounts and random shipping loss.

	Demand fluctuation measurement	
	(HAPPO, PPO, non-stationary base stock)	
Experiment settings	Average	Amplification (%)
Price discounts	(0.774, 0.849, 0.583)	(100.9, 121.4, 27.27)
Random shipping loss	(0.633, 0.697, 0.577)	(50.22, 75.99, 30.02)

HAPPO = heterogeneous-agent proximal policy optimization; PPO = proximal policy optimization.

## 4. MARL in Supply Chain Network System

In this section, we consider the multi-echelon inventory management problem in a supply chain network system. With little modification, MADRL can be easily adapted to the supply chain network system.

### 4.1. Model Formulation in Supply Chain Network System

**Figure 15.**
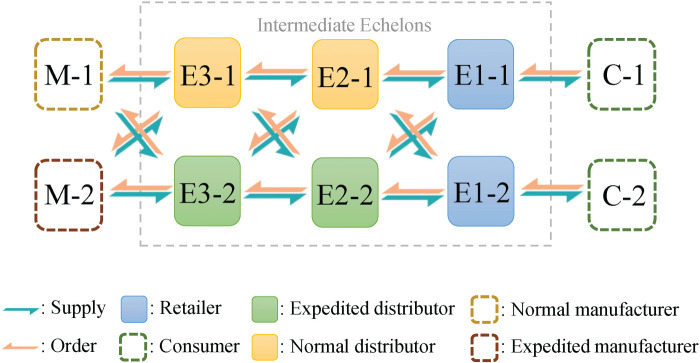
A supply chain network system with three echelons.

We study the supply chain network system with M intermediate echelons, and each echelon contains two actors. An example with M=3 is shown in Figure 15. Actors are homogeneous in the echelon that directly satisfies customer demand and heterogeneous in other echelons. In each echelon with heterogeneous actors, similar to the dual-sourcing inventory management problem, one actor is taken as a normal supply source and the other as an expedited supply source with a higher selling price and a shorter lead time.

We use a 2-tuple (i,j) to represent the j -th actor in the i -th echelon. For an echelon with heterogeneous actors, we make the first actor the normal source with lead time $L^{1}$ and the second actor the expedited source with lead time $L^{2}$ , where $L^{1}>L^{2}$ . In each period, each actor receives two orders from its two downstream actors and places two orders with its two upstream actors. We use $O_{t}^{i,j,k}$ to denote the quantity of orders placed by actor (i,j) to actor (i+1,k) , use $B_{t}^{i,j,k}$ to denote actor (i,j) ’s backlogs to actor (i−1,k) (the accumulative unmet demand of actor (i−1,k) ), and use $I_{t}^{i,j}$ to denote actor (i,j) ’s inventory. We use $c^{i,j,k},\;b^{i,j}$ , and $h^{i,j}$ to denote actor (i,j) ’s ordering cost coefficient when ordering from actor (i+1,k) , actor (i,j) ’s backlog cost coefficient and actor (i,j) ’s holding cost coefficient, respectively.

**Figure 16.**
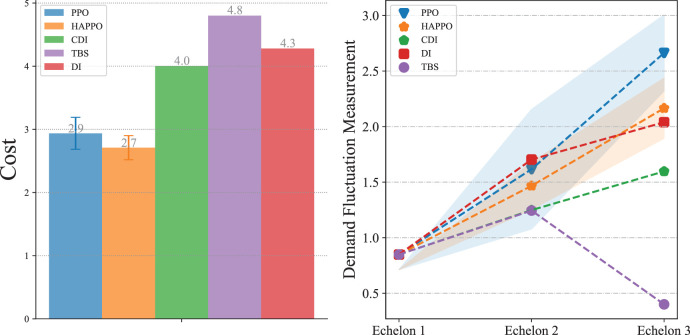
Comparison of system’s overall costs and the bullwhip effect in the supply chain network system.

Similar to the serial supply chain system, the actor’s ordering is constrained by the inventory state of its upstream suppliers. In each period t , each actor (i,j) faces total orders of $\sum_{k=1}^{2}(O_{t}^{i-1,k,j}+B_{t}^{i,j,k})$ from its two downstream actors. We use $P_{t}^{i,j,k}$ to represent the quantity of shipment from actor (i,j) to actor (i−1,k) in period t . The inventory update for actor (i,j) is then given by the following equation:

$$I_{t+1}^{i,j}={\left(I_{t}^{i,j}+\sum_{k=1}^{2}P_{t-L^{k}}^{i+1,k,j}-\sum_{k=1}^{2}(O_{t}^{i-1,k,j}+B_{t}^{i,j,k})\right)}^{+}.$$

Since each actor (i,j) now faces two downstream actors, we assume that it first fulfills orders from the expedited-source actor (i−1,2) and then satisfies orders from the normal-source actor (i−1,1) . The quantity of shipment $P_{t}^{i,j,2}$ from actor (i,j) to actor (i−1,2) is given by the following equation:

$$P_{t}^{i,j,2}=\min\{I_{t}^{i,j}+\sum_{k=1}^{2}P_{t-L^{k}}^{i+1,k,j},O_{t}^{i-1,2,j}+B_{t}^{i,j,2}\}.$$

The quantity of shipment $P_{t}^{i,j,1}$ from actor (i,j) to actor (i−1,1) is then given by the following equation:

$$P_{t}^{i,j,1}=\min\{O_{t}^{i-1,1,j}+B_{t}^{i,j,1},I_{t}^{i,j}+\sum_{k=1}^{2}P_{t-L^{k}}^{i+1,k,j}-P_{t}^{i,j,2}\}.$$

The update of backlog for actor (i,j) is given by the following equation:

$$B_{t+1}^{i,j,k}=B_{t}^{i,j,k}+O_{t}^{i-1,k,j}-P_{t}^{i,j,k},\;\forall k\in \left\{1,2\right\}.$$

The costs for actor (i,j) in period t are given by the following equation:

$$C_{t}^{i,j}=h^{i,j}I_{t}^{i,j}+b^{i,j}\sum_{k=1}^{2}B_{t}^{i,j,k}+\sum_{k=1}^{2}c^{i,j,k}P_{t}^{i+1,k,j}.$$

The overall costs of the supply chain network in period t is given by the following equation:

(11) $$C_{t}^{\text{total}}=\sum_{i=1}^{M}\sum_{j=1}^{2}C_{t}^{i,j}.$$

We formulate the supply chain network system as a POMG. The action space for actor (i,j) is { $\left(1,1),\ldots ,(1,O^{\max}),\ldots ,(O^{\max},O^{\max}\right)$ }, where the 2-tuple represents quantities of orders from the normal source and the expedited source.

To include the information on inventory, backlogs, historical demand, and in-transit orders, actor (i,j) ’s observation is given by the following equation:

$$\begin{array}{rl} o_{t}^{i,j} & =(I_{t}^{i,j},\sum_{k=1}^{2}B_{t}^{i,j,k},\sum_{k=1}^{2}O_{t-1}^{i-1,k,j},P_{t-L^{1}}^{i+1,1,j},\ldots ,P_{t-L^{2}-1}^{i+1,1,j},\\ & \;\sum_{k=1}^{2}P_{t-L^{2}}^{i+1,k,j},\ldots ,\sum_{k=1}^{2}P_{t-1}^{i+1,k,j}). \end{array}$$

Similar to the serial supply chain system, we define the reward function for actor (i,j) under this scenario as a combination of its own costs and the system’s overall costs, which is given by the following equation:

$$r_{t}^{i,j}=-\alpha C_{t}^{i,j}-(1-\alpha )C_{t}^{\text{total}}.$$

We also conduct controlled experiments on α , and the results are shown in Appendix H. Similar to the Observation 1 from the serial supply chain system, the best performing α that achieves the lowest overall costs for the supply chain network system is between 0 and 1 ( 0.625 , to be specific). Note that Propositions 1 and 2 also hold for the supply chain network system. The aforementioned results once again verify our analysis of the variance-bias trade-off introduced in Section 3.1. Based on the results, we determine the value for α as 0.625 for other experiments conducted for the supply chain network system.

### 4.2. Baseline Policies and Experiment Settings

Similar to Section 3.3, we construct both the PPO baseline and several heuristic policies to evaluate HAPPO’s performance in the supply chain network system. Specifically, we choose three heuristic policies for the dual-sourcing inventory management problem, that is, DI, CDI, and TBS, whose implementation is provided in Appendix F.

For the experiment settings, we compare the overall costs of the system and the bullwhip effect for different policies in a typical supply chain network system with M=3 and Merton customer demand. The backlog cost coefficients and holding cost coefficients are set to 1 for all actors. The ordering cost coefficients are set to 1 when ordering from the expedited source and 0.5 when ordering from the normal source for all actors. The lead time is 4 for the normal source and 2 for the expedited source. We measure the demand fluctuation of echelon i as follows: $\frac{1}{2}\sum_{j=1}^{2}\sqrt{\sum_{t=0}^{T}(\sum_{k=1}^{2}O_{t}^{i-1,k,j}-{\bar{O}}^{i-1,j}{)}^{2}}/(\sqrt{T+1}\;{\bar{O}}^{i-1,j}),$ where ${\bar{O}}^{i-1,j}=\frac{1}{T+1}\sum_{t=0}^{T}\sum_{k=1}^{2}O_{t}^{i-1,k,j}$ and T is the number of simulated periods for one episode.

### 4.3. Numerical Results

As shown in Figure 16, HAPPO achieves the lowest overall costs among all the policies we consider. Also, HAPPO leads to a less significant bullwhip effect compared to the PPO baseline. The results indicate that with a more complex supply chain structure, HAPPO is still superior in reducing the costs of the system and effective in alleviating the bullwhip effect. We provide the detailed policy visualization in the supply chain network system in Appendix I.

## 5. Conclusions

In this article, we demonstrate the superiority of MADRL in multi-echelon inventory management problems with limited information sharing, a complex supply chain structure, and non-stationary market environments. In both a serial supply chain system and a supply chain network system, policies constructed by HAPPO achieve the lowest overall costs among policies constructed by PPO, a single-agent DRL method, and other heuristic policies. Also, policies constructed by HAPPO yield a smaller bullwhip effect than policies constructed by PPO, which indicates the advantage of the upfront-only information-sharing mechanism used in MADRL. As the main takeaway of applying HAPPO in multi-echelon inventory management, compared to setting the minimization target of each actor as the overall costs of the system, HAPPO achieves lower overall costs when each actor learns to minimize a combination of its own costs and overall costs of the system.

In principle, MADRL can be applied to any multi-echelon inventory management problem as long as it can be formulated as a POMG. Other than HAPPO, there also exist some other effective MADRL algorithms following the CTDE scheme, such as Lowe et al. (2017), which can be used to tackle various multi-echelon inventory management problems where information sharing among actors is somehow restricted.

We provide extensible codes for applying HAPPO to any customized multi-echelon inventory management problem at https://github.com/xiaotianliu01/Multi-Agent-Deep-Reinforcement-Learning-on-Multi-Echelon-Inventory-Management which can serve as a base for quick implementation and future research.

## Supplemental Material

sj-pdf-1-pao-10.1177_10591478241305863 - Supplemental material for Multi-Agent Deep Reinforcement Learning for Multi-Echelon Inventory Management Supplemental material, sj-pdf-1-pao-10.1177_10591478241305863 for Multi-Agent Deep Reinforcement Learning for Multi-Echelon Inventory Management by Xiaotian Liu, Ming Hu, Yijie Peng and Yaodong Yang in Production and Operations Management

## Notes

1. The more frequently used notation by the previous literature such as Oroojlooyjadid et al. (2022) is to denote the on-hand inventory by $(I_{t}^{i}{)}^{+}$ and denote backlogs by $(I_{t}^{i}{)}^{-}$ , where $x^{+}$ represents max(x,0) and $x^{-}$ represents max(−x,0) . For the convenience of formula expressions in this article, we adopt a different notation by using $I_{t}^{i}\geq 0$ to represent the on-hand inventory and introduce a new symbol $B_{t}^{i}\geq 0$ to represent backlogs.
