# Supplementary material for: Multi-Agent Deep Reinforcement Learning for Multi-Echelon Inventory Management
Source: Prod Oper Manag. 2024 Dec 29;34(7):1836–56. doi: 10.1177/10591478241305863 (PMC13044432; doi:10.1177/10591478241305863)
Supplement: sj-pdf-1-pao-10.1177_10591478241305863 - Supplemental material for Multi-Agent Deep Reinforcement Learning for Multi-Echelon Inventory Management [file sj-pdf-1-pao-10.1177_10591478241305863.pdf]

E-Companion for "Multi-Agent Deep Reinforcement Learning for Multi-Echelon Inventory Management"

A Proof of Proposition

Proof of Proposition 1. By the definition (7) of reward, for any actor $i \in \{1, \dots, M\}$, we have

$$\begin{aligned}
\left|\mathbb{E}\left[\sum_{t=0}^{\infty}\gamma^t r_t^i\right] - \mathbb{E}\left[-\sum_{t=0}^{\infty}\gamma^t C_t^{\text{total}}\right]\right| &= \left|\mathbb{E}\left[\sum_{t=0}^{\infty}\gamma^t\big(-\alpha C_t^i - (1-\alpha)C_t^{\text{total}}\big)\right] - \mathbb{E}\left[-\sum_{t=0}^{\infty}\gamma^t C_t^{\text{total}}\right]\right| \\
&= \left|\mathbb{E}\left[\sum_{t=0}^{\infty}\gamma^t\big(-\alpha C_t^i + \alpha C_t^{\text{total}}\big)\right]\right| \\
&= \alpha\left|\mathbb{E}\left[\sum_{t=0}^{\infty}\gamma^t\big(-C_t^i + C_t^{\text{total}}\big)\right]\right|,
\end{aligned}$$

where the last equation follows from the fact that $\alpha \in [0,1]$. With the joint policy unchanged, the expectation term $\mathbb{E}\big[\sum_{t=0}^{\infty}\gamma^t\big(-C_t^i + C_t^{\text{total}}\big)\big]$ is a constant. Hence, the considered bias $\big|\mathbb{E}\big[\sum_{t=0}^{\infty}\gamma^t r_t^i\big] - \mathbb{E}\big[-\sum_{t=0}^{\infty}\gamma^t C_t^{\text{total}}\big]\big|$ for any actor $i \in \{1, \dots, M\}$ is monotone increasing w.r.t. the parameter α and is 0 when $\alpha = 0$. ■

Proof of Proposition 2. By the definition (7) of reward, for any actor $i \in \{1, \dots, M\}$, we have

$$\begin{aligned}
\text{Var}\left(\sum_{t=0}^{\infty}\gamma^t r_t^i\right) &= \text{Var}\left(\sum_{t=0}^{\infty}\gamma^t\big(-\alpha C_t^i - (1-\alpha)C_t^{\text{total}}\big)\right) \\
&= \text{Var}\left(\sum_{t=0}^{\infty}\gamma^t\Big(-C_t^i - (1-\alpha)\sum_{j\in\{1,\dots,M\}/\{i\}} C_t^j\Big)\right) \\
&= \text{Var}\left(\sum_{t=0}^{\infty}\gamma^t C_t^i\right) + (1-\alpha)^2\text{Var}\left(\sum_{t=0}^{\infty}\sum_{j\in\{1,\dots,M\}/\{i\}}\gamma^t C_t^j\right) + 2(1-\alpha)\text{Cov}\left(\sum_{t=0}^{\infty}\gamma^t C_t^i, \sum_{t=0}^{\infty}\sum_{j\in\{1,\dots,M\}/\{i\}}\gamma^t C_t^j\right).
\end{aligned}$$

Hence, the variance $\text{Var}\big(\sum_{t=0}^{\infty}\gamma^t r_t^i\big)$ is a quadratic equation w.r.t. the variable α. It can be easily seen that when $\text{Cov}\big(\sum_{t=0}^{\infty}\gamma^t C_t^i, \sum_{t=1}^{\infty}\sum_{j\in\{1,\dots,M\}/\{i\}}\gamma^t C_t^j\big) \geq 0$, the variance $\text{Var}\big(\sum_{t=0}^{\infty}\gamma^t r_t^i\big)$ is monotone decreasing w.r.t. the parameter $\alpha \in [0,1]$. ■

Proof of Proposition 3. By reward function (7), when $\alpha = 0$, all actors receive the same reward as

$$r_t^i = \mathcal{R}^i(a_t^i|s_t) = -\alpha C_t^i - (1-\alpha)C_t^{\text{total}} = -C_t^{\text{total}}. \tag{A.1}$$

Hence, the POMG under this scenario is a fully cooperative game where the joint reward is the opposite of the overall costs of the system $-C_t^{\text{total}}$.

By Theorem 2 in Kuba et al. (2021), when applying HAPPO to a fully cooperative POMG, a sequence $(\boldsymbol{\pi}_k)_{k=0}^{\infty}, \boldsymbol{\pi}_k = (\pi_k^1, \dots, \pi_k^N)$ of joint policies has the monotonic improvement property as

$$\mathbb{E}_{\boldsymbol{a}_{0:\infty}\sim\boldsymbol{\pi}_{k+1}, s_{0:\infty}\sim\mathcal{T}}\left[\sum_{t=0}^{\infty}\gamma^t r_t\right] \geq \mathbb{E}_{\boldsymbol{a}_{0:\infty}\sim\boldsymbol{\pi}_k, s_{0:\infty}\sim\mathcal{T}}\left[\sum_{t=0}^{\infty}\gamma^t r_t\right]. \tag{A.2}$$

HAPPO's monotonic improvement property (A.2) is derived mainly based on the monotonic improvement property of PPO introduced by Schulman et al. (2017). Schulman et al. (2017) prove that the performance of PPO, a single-agent reinforcement learning algorithm, can be improved monotonically along with

training. To apply PPO to Multi-Agent Systems (MAS) developed from fully cooperative Markov Games and maintain PPO's monotonic improvement property, Kuba et al. (2021) make a modification to PPO and introduce HAPPO. Kuba et al. (2021) show that simply equipping each actor in MAS with a PPO model has no guarantee for the monotonical improvement of actors' policies. Hence, Kuba et al. (2021) introduce a sequential policy update scheme where actors sequentially update their policies constructed by PPO with reference to other actors' updated policies and prove that HAPPO achieves a monotonic improvement as shown in (A.2) with the proposed sequential policy update scheme. More details about the proof of (A.2) can be found in Kuba et al. (2021).

Replacing r_t in equation (A.2) with $-C_t^{\text{total}}$ in (A.1) concludes the proof for Proposition 3. ■

Proof of Proposition 4. Assume that each actor now takes the optimal policy as

$$O_t^i = O_t^0,\ \forall i \in \{1,\ldots,M\},\ \forall t \geq 0 \tag{A.3}$$

For the most upstream actor M in period 0, since the lead time is 0, by equation (1) we have

$$P_0^M = \min\left\{O_0^{M-1} + B_0^M, I_0^M + P_0^{M+1}\right\}. \tag{A.4}$$

With $I_0^M = 0$ and $B_0^M = 0$ assumed by conditions, we have

$$P_0^M = \min\left\{O_0^{M-1}, P_0^{M+1}\right\}. \tag{A.5}$$

With equation (2), we have

$$P_0^{M+1} = O_0^M. \tag{A.6}$$

Hence we have

$$P_0^M = \min\left\{O_0^{M-1}, O_0^M\right\}. \tag{A.7}$$

With actor M and actor $M-1$ following the optimal policy in (A.3), we have

$$P_0^M = O_0^0. \tag{A.8}$$

By the inventory update equation (4), we have

$$I_1^M = I_0^M + P_0^{M+1} - P_0^M = 0. \tag{A.9}$$

By the backlog update equation (3), we have

$$B_1^M = B_0^M + O_0^{M-1} - P_0^M = 0. \tag{A.10}$$

By equation (5), the costs for actor M in period 0 are then given by

$$C_0^M = h^M I_0^M + b^M B_0^M = 0. \tag{A.11}$$

By sequentially applying the same analysis illustrated above on actors $\{M-1,\ldots,1\}$ in period 0, we can get

$$C_0^i = 0,\ \forall i \in \{1,\ldots,M\}, \tag{A.12}$$

and

$$B_1^i = I_1^i = 0,\ \forall i \in \{1,\ldots,M\}. \tag{A.13}$$

Equation (A.13) shows that after period 0, each actor still has zero inventory and zero backlog. Hence, the above-mentioned analysis also holds for periods $t \geq 1$. Therefore, when all actors take the policy in (A.3), the long-term overall costs of the system are given by

$$\sum_{t=0}^{\infty} \gamma C_t^{\text{total}} = \sum_{t=0}^{\infty} \gamma \sum_{i=1}^{M} C_t^i = \sum_{t=0}^{\infty} \gamma \sum_{i=1}^{M} 0 = 0. \tag{A.14}$$

Hence policy in (A.3) is the optimal policy that minimizes the overall costs of the system. ■

B Statistics of Real-life Demand Data

We select real-life demands of 20 retailing goods provided by Walmart in the M5 forecasting competition (Howard et al. 2020). The length of each demand trace is 1941. For each trace, we select the first 1741 demand points to conduct model training and the last 200 demand points to conduct model evaluation. The statistics of the selected real-life demands are provided in Table B.1.

Table B.1 Statistics of selected real-life demand data.

Item_id	Store_id	State_id	Mean	Standard Deviation	Median
HOBBIES_1_134	CA_1	CA	4.154044	3.778716	4
HOBBIES_1_147	CA_1	CA	4.210716	3.823435	4
HOUSEHOLD_1_118	CA_1	CA	7.646574	5.814829	6
FOODS_1_018	CA_1	CA	9.691396	6.532215	9
FOODS_1_046	CA_1	CA	7.12983	5.745203	6
FOODS_1_086	CA_1	CA	5.514683	4.443174	5
FOODS_2_013	CA_1	CA	4.110252	2.794379	4
FOODS_2_181	CA_1	CA	6.303452	3.809494	6
FOODS_2_371	CA_1	CA	8.230294	4.37689	8
FOODS_3_226	CA_1	CA	6.979907	3.791869	6
FOODS_3_228	CA_1	CA	8.992272	4.537717	8
FOODS_3_473	CA_1	CA	8.750129	5.043848	8
FOODS_3_377	CA_1	CA	11.08604	4.750044	11
FOODS_3_668	CA_1	CA	10.3493	4.576398	10
HOUSEHOLD_1_179	CA_3	CA	11.77383	9.333528	11
FOODS_2_128	CA_3	CA	10.95981	10.31525	9
FOODS_3_348	CA_3	CA	12.11592	8.536521	12
FOODS_3_458	CA_3	CA	12.76404	6.321212	12
FOODS_3_697	CA_1	CA	8.050489	4.317813	8
HOUSEHOLD_1_083	CA_2	CA	6.083977	4.423912	5

C Pseudocode for Training Process of HAPPO

Algorithm 1 HAPPO for Multi-Echelon Inventory Management Problems

Input: Number of actors M, episodes K, periods per episode T, discount factor γ, clip parameter ε, entropy coefficient β_E.

Initialize: Actor networks $(\pi_0^1, \ldots, \pi_0^M)$ and critic networks $(V_0^1, \ldots, V_0^M)$.

for $k = 0, 1, \ldots, K-1$ **do**

  Get initial observations for all actors $(o_0^1, \ldots, o_0^M)$

  for $t = 0, 1, \ldots, T$ **do**

    Apply actors' policies $(\pi_k^1, \ldots, \pi_k^M)$ on current observations $(o_t^1, \ldots, o_t^M)$ and sample actions $(a_t^1, \ldots, a_t^M)$ from probability distributions of actions $\left(\pi_k^1(\cdot|o_t^1), \ldots, \pi_k^M(\cdot|o_t^M)\right)$

    Execute actions $(a_t^1, \ldots, a_t^M)$, observe rewards $(r_t^1, \ldots, r_t^M)$ and next observations $(o_{t+1}^1, \ldots, o_{t+1}^M)$

    Record transitions $(o_t^1, a_t^1, r_t^1, o_{t+1}^1, \ldots, o_t^M, a_t^M, r_t^M, o_{t+1}^M)$

  end for

  Compute advantage A_t^i for actor $i \in \{1, \ldots, M\}$ in period $t \in \{0, \ldots, T\}$ using its critic network V_k^i:

$$A_t^i = \sum_{l=0}^{T-t} \gamma^l r_{t+l}^i + \gamma^{T+1-t} V_k^i \left(o_{T+1}^1, \ldots, o_{T+1}^M\right) - V_k^i \left(o_t^1, \ldots, o_t^M\right)$$

  Draw a random permutation of actors $i_{1:M}$

  Initialize sequential update factor $F_t^{i_1} = 1, \ \forall t \in \{0, \ldots, T\}$ for actor i_1

  for actor $i_m = i_1, \ldots, i_M$ **do**

    Compute policy loss for actor i_m:

$$\text{Policy loss} = - \frac{1}{T+1} \sum_{t=0}^{T} \min \left\{ \frac{\pi_k^{i_m}\left(a_t^{i_m}|o_t^{i_m}\right)}{\pi_{k-1}^{i_m}\left(a_t^{i_m}\middle|o_t^{i_m}\right)} F_t^{i_m} A_t^{i_m}, \text{clip} \left(\frac{\pi_k^{i_m}\left(a_t^{i_m}|o_t^{i_m}\right)}{\pi_{k-1}^{i_m}\left(a_t^{i_m}\middle|o_t^{i_m}\right)}, 1-\varepsilon, 1+\varepsilon \right) F_t^{i_m} A_t^{i_m} \right\}$$

    Compute entropy loss for actor i_m:

$$\text{Entropy Loss} = \sum_{t=0}^{T} \pi_k^{i_m}\left(\cdot|o_t^{i_m}\right) \log\left(\pi_k^{i_m}\left(\cdot|o_t^{i_m}\right)\right)$$

    Update parameters for actor i_m's actor network from $\pi_k^{i_m}$ to $\pi_{k+1}^{i_m}$ by minimizing Policy loss + β_EEntropy Loss with Adam optimizer (Kingma and Ba 2014)

    Compute sequential update factor $F_t^{i_{m+1}}$ for actor i_{m+1}:

$$F_t^{i_{m+1}} = F_t^{i_m} \frac{\pi_{k+1}^{i_m}\left(a_t^{i_m}|o_t^{i_m}\right)}{\pi_k^{i_m}\left(a_t^{i_m}\middle|o_t^{i_m}\right)}, \forall t \in \{0, \ldots, T\}$$

    Compute critic loss for actor i_m:

$$\text{Critic Loss} = \frac{1}{T+1} \sum_{t=0}^{T} \left(V_k^{i_m}(o_t^1, \ldots, o_t^M) - \sum_{\tau=0}^{T-t} \gamma^\tau r_{t+\tau}^{i_m} - \gamma^{T-t+1} V_k^{i_m}(o_{T+1}^1, \ldots, o_{T+1}^M) \right)^2$$

    Update actor i_m's critic network parameters from $V_k^{i_m}$ to $V_{k+1}^{i_m}$ by minimizing Critic Loss with Adam optimizer

  end for

end for

D Implementation of Non-Stationary Base Stock and Non-Stationary (s, S) policies.

Non-stationary base stock policy. We adopt the method in Neale and Willems (2009) which is based on the guaranteed service modeling framework. In the method of Neale and Willems (2009), the base stock level changes by referring to the mean and variance of historical demands. Specifically, we first compute the rolling mean $\bar{D}_t^i$ and variance $\tilde{D}_t^i$ of historical demands for actor $i \in \{1, \ldots, M\}$ at period $t \in \{0, \ldots, T\}$ as

$$\begin{aligned} \bar{D}_t^i &= \frac{1}{H} \sum_{\tau=t-H}^{t-1} O_\tau^{i-1}, \\ \tilde{D}_t^i &= \frac{1}{H} \sum_{\tau=t-H}^{t-1} (O_\tau^{i-1} - \bar{D}_t^i)^2, \end{aligned} \tag{D.1}$$

where H is the length of the historical demands used to compute the statistics. By experiments, we find that setting H to 40 under our setting of $T = 200$ contributes to the best performance of the base stock policy. After obtaining the historical mean and variance for demands, the base stock level for actor $i \in \{1, \ldots, M\}$ at period $t \in \{0, \ldots, T\}$ is computed by

$$\text{Base Stock Level} = L\bar{D}_t^i + z\sqrt{L\tilde{D}_t^i},$$

where z is a parameter that reflects the service level of the system. To determine the parameter z, we try different choices of z in a wide range and choose the one that achieves the lowest overall costs on the test demand data. An example of the results for enumerating z under the setting of Merton demands, 3 echelons, and no fixed cost is shown in Figure D.1.

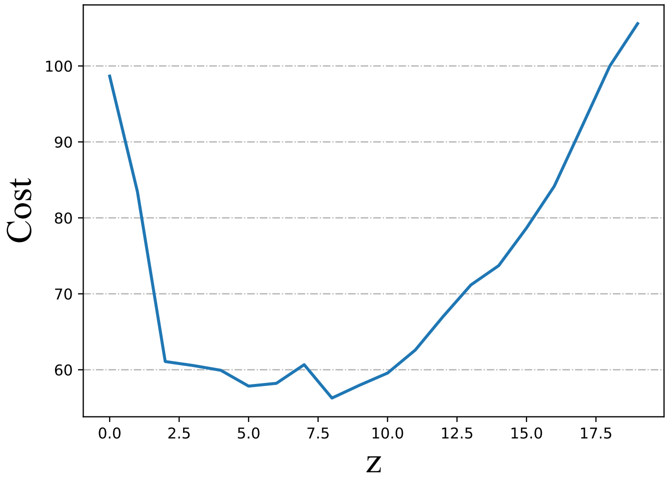

Figure D.1 Results of enumerating parameter z.

In the case of Figure D.1, z is determined as 8 which achieves the lowest costs of the system.

Non-stationary (s, S) policy. For non-stationary (s, S) policy, we adopt the method in Bollapragada and Morton (1999). Specifically, we first compute the optimal policy parameters, i.e., s and S, under the case of i.i.d. Poisson demands by using the algorithm in Zheng and Federgruen (1991). The s and S parameters computed using the algorithm in Zheng and Federgruen (1991) under our experiment setting are shown in Table D.1.

Table D.1 Optimal parameters for stationary problem computed by algorithm in Zheng and Federgruen (1991).

**Demand Mean**	10	12	14	16	18	20	22	24	26	28	30	32	34	36	38	40
s	0	2	4	6	8	10	12	14	16	18	20	22	24	26	28	30
S	10	12	14	16	18	20	22	24	26	28	30	32	34	36	38	40
**Demand Mean**	42	44	46	48	50	52	54	56	58	60	62	64	66	68	70	72
s	32	34	36	38	40	42	44	46	48	50	52	54	56	58	60	62
S	42	44	46	48	50	52	54	56	58	60	62	64	66	68	70	72

For each actor $i \in \{1, \ldots, M\}$ at period $t \in \{0, \ldots, T\}$, we compute the mean $\bar{D}_t^i$ of historical demands by using equation (D.1). Then we look up Table D.1 and take the optimal policy parameters s and S associated with i.i.d. Poisson demands with mean $L\bar{D}_t^i$ as parameters for non-stationary (s, S) policy at period t. By experiments, we find the best choice of H, which is the length of the historical demands, is 1 under the non-stationary (s, S) setting.

E Computation of Optimal Base Stock Level

In a serial supply chain system where demands are i.i.d. and backlog costs only occur at the most downstream stage, the optimal policy is the base stock policy (Clark and Scarf 1960). We compute the optimal base stock level by following the method introduced by Shang and Song (2003). For each actor $i \in \{1, \ldots, M\}$, let $\mathcal{D}_i(\cdot)$ be the distribution function of $L\sum_{j=0}^{i-1} O_t^j$, which is the total leadtime demand in the subsystem consisting stages 1 through i. By assumption, the external demand O_t^0 for any period t is an i.i.d. Poisson distributed random variable. Hence, when applying the base stock policy, the random variable $L\sum_{j=0}^{i-1} O_t^j$ for any actor i is also Poisson distributed. Let U_i be the optimal echelon base stock level of stage i and U_i^* be the optimal installation base stock level of actor i. Then the lower bound $\underline{U}_i$ for the optimal echelon base stock level U_i is given by

$$\underline{U}_i = \mathcal{D}_i^{-1}\left(\frac{b^1 + \sum_{j=i+1}^{M} h^j}{b^1 + \sum_{j=1}^{M} h^j}\right).$$

The upper bound $\overline{U}_i$ for the optimal echelon base stock level U_i is given by

$$\overline{U}_i = \mathcal{D}_i^{-1}\left(\frac{b^1 + \sum_{j=i+1}^{M} h^j}{b^1 + \sum_{j=i}^{M} h^j}\right).$$

After getting the optimal echelon base stock levels, the optimal installation base stock level U_i^* of actor i can be computed by $U_i^* = U_i - U_{i-1}$ with $U_0 = 0$. Since we only have the upper and lower bounds of optimal

echelon base stock level U_i for each actor i, we determine U_i by running numerical evaluation under all possible values and selecting the value with the lowest overall costs. Under the setting of $L = 2$, the bounds for optimal echelon base stock levels are $\underline{U}_1 = 11$, $\overline{U}_1 = 12$, $\underline{U}_2 = 19$, $\overline{U}_2 = 22$, $\underline{U}_3 = 25$, and $\overline{U}_3 = 30$. By searching among these ranges, the optimal echelon base stock levels are determined as $U_1 = 11$, $U_2 = 22$, and $U_3 = 29$, which makes the optimal installation base stock levels $U_1^* = 11$, $U_2^* = 11$, and $U_3^* = 7$. Under the setting of $L = 4$, the bounds for optimal echelon base stock levels are $\underline{U}_1 = 22$, $\overline{U}_1 = 23$, $\underline{U}_2 = 39$, $\overline{U}_2 = 43$, $\underline{U}_3 = 54$, and $\overline{U}_3 = 60$. By searching among these ranges, the optimal echelon base stock levels are determined as $U_1 = 22$, $U_2 = 43$, and $U_3 = 59$, which makes the optimal installation base stock levels $U_1^* = 22$, $U_2^* = 21$, and $U_3^* = 16$.

F Implementation of Baselines for Supply Chain Network

In our experiments conducted on the supply chain network, we adopt three baselines, i.e., DI, CDI, and TBS. In DI, ordering from both the normal source and expedited source follows the base stock policy. It has two parameters, i.e., two base stock levels for two sources. CDI further complicates DI by adding a constant ordering limit or "cap" on the normal source, i.e., ordering from the normal source can not exceed this limit. It has three parameters, i.e., two base stock levels for two sources and the ordering limit for the normal source. TBS makes constant orders from the normal source and follows an (s, S) policy when ordering from the expedited source. It has three parameters, i.e., the constant ordering quantity for the normal source and the s, S parameters for the expedited source. To determine the best-performing parameters for each baseline, we experiment with all possible combinations of parameters in a wide range and select the parameter setting that achieves the lowest overall costs on the evaluation data.

G Exogenous Parameters

Table G.1 Hypherparameters in HAPPO.

Hyperparameter	Values
Length T of one episode	200
Number of layers for GRU	2
Width for GRU	128
Discount factor γ	0.95
Learning rate	1e-4
Clip parameter ε	0.2
Entropy Coefficient β_E	0.01

Note. *All hyperparameters listed above are the same in the actor network and critic network.*

Table G.2 Exogenous coefficients in demand generation.

Exogenous Coefficients	Serial Supply Chain System	Supply Chain Network System
μ	5e-5	5e-5
σ	1e-2	1e-2
λ	15	15
ℓ_1	10	3
ℓ_2	0	0
ℓ_3	0.01	0.01

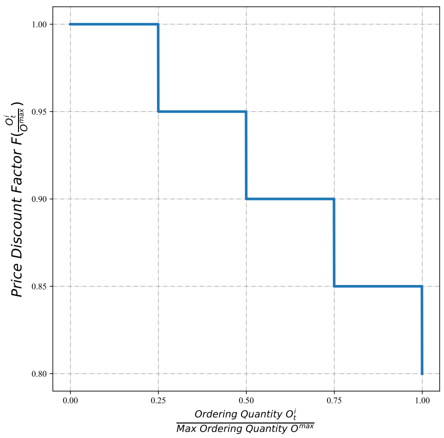

Figure G.1 Price discount factor for the serial supply chain system with price discounts.

H Effect of α on Overall Costs of Supply Chain Network System

Results of the controlled experiments on α are shown in Figure H.1. Based on these results, we set α as 0.625 for all experiments conducted on the supply chain network system.

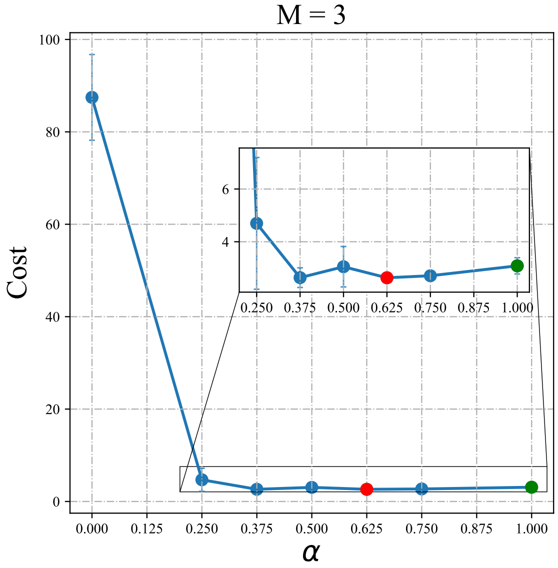

Note. *The red point represents the optimal overall costs achieved by* α *between 0 and 1, and the green point represents the near-optimal overall costs achieved by* $\alpha = 1$.

Figure H.1 Effect of α on overall costs of the supply chain network.

I Policy Visualizations

We record and visualize demand, orders, and inventory for each actor in the system for 60 periods. The visualization in the typical serial supply chain system with 3 echelons and without fixed costs is provided in Figure I.2. The visualization in the typical supply chain network system is provided in Figure I.1.

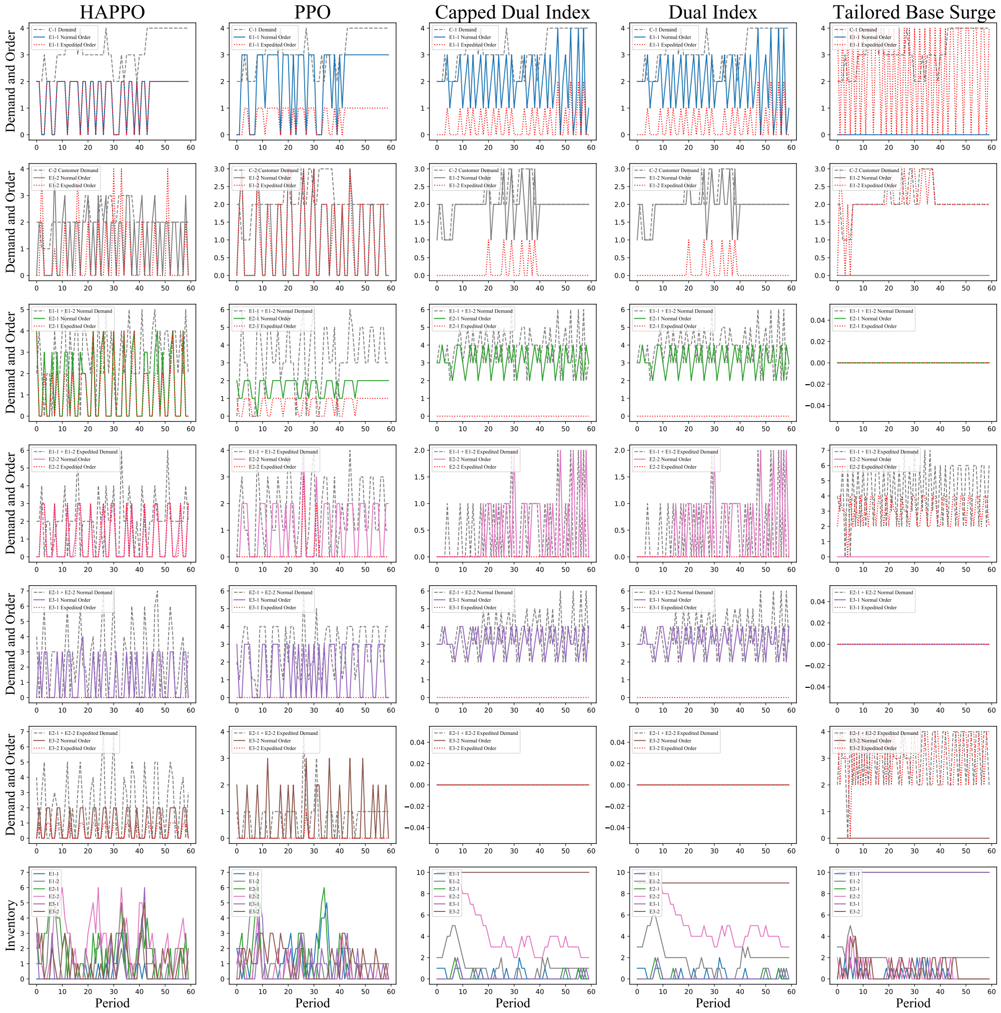

Figure I.1 Policy visualization for the supply chain network system.

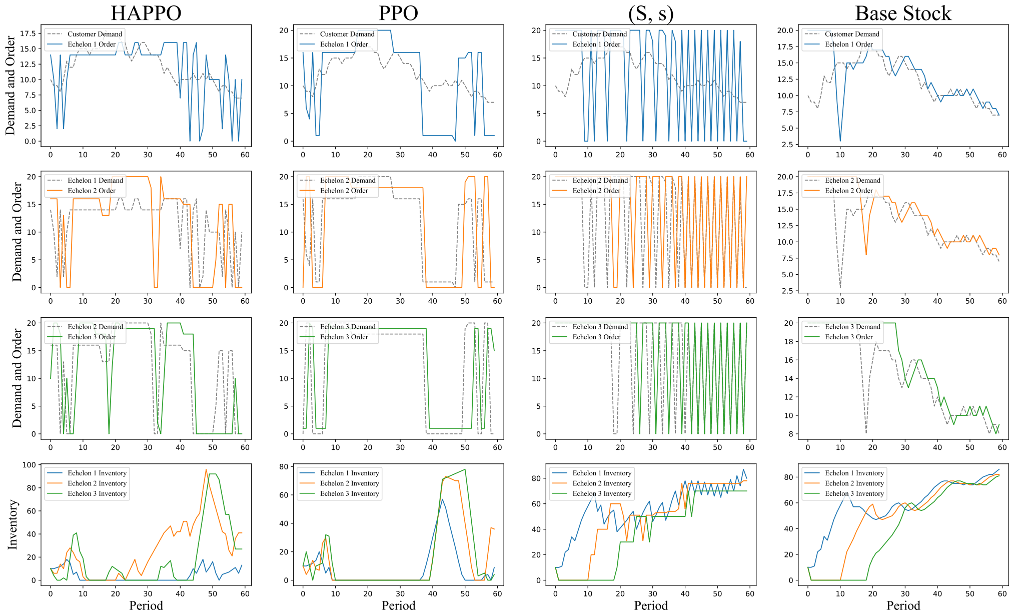

Figure I.2 Policy visualization for the serial supply chain system.

References

Bollapragada, S, TE Morton. 1999. A simple heuristic for computing nonstationary (s, s) policies. *Oper. Res.*, 47 (4), 576-584.

Clark, AJ, H Scarf. 1960. Optimal policies for a multi-echelon inventory problem. *Manag. Sci.*, 6 (4), 475-490.

Howard, A, inversion, S Makridakis, vangelis. 2020. M5 forecasting - accuracy. URL https://kaggle.com/competitions/m5-forecasting-accuracy.

Kingma, DP, J Ba. 2014. Adam: A method for stochastic optimization., URL https://arxiv.org/abs/1412.6980.

Kuba, JG, R Chen, M Wen, Y Wen, F Sun, J Wang, Y Yang. 2021. Trust region policy optimisation in multi-agent reinforcement learning, URL https://arxiv.org/abs/2109.11251.

Neale, JJ, SP Willems. 2009. Managing inventory in supply chains with nonstationary demand. *Interfaces*, 39 (5), 388-399.

Schulman, J, F Wolski, P Dhariwal, A Radford, O Klimov. 2017. Proximal policy optimization algorithms, URL http://arxiv.org/abs/1707.06347.

Shang, KH, JS Song. 2003. Newsvendor bounds and heuristic for optimal policies in serial supply chains. *Manag. Sci.*, 49 (5), 618-638.

Zheng, YS, A Federgruen. 1991. Finding optimal (s, s) policies is about as simple as evaluating a single policy. *Ope. Res.*, 39 (4), 654-665.
